# Supplementary material for: Large-language models facilitate discovery of the molecular signatures regulating sleep and activity
Source: Nat Commun. 2024 May 1;15:3685. doi: 10.1038/s41467-024-48005-w (PMC11063160; doi:10.1038/s41467-024-48005-w)
Supplement: Supplementary file 1 — Supplementary Information [file 41467_2024_48005_MOESM1_ESM.pdf]

Supplementary Information

Large-language models facilitate discovery of the molecular signatures regulating sleep and activity

Di Peng[1,†], Liubin Zheng[1,†], Dan Liu[1,†], Cheng Han[1,†], Xin Wang[1], Yan Yang[1], Li Song[1], Miaoying Zhao[1], Yanfeng Wei[1], Jiayi Li[1], Xiaoxue Ye[1], Yuxiang Wei[1], Zihao Feng[1], Xinhe Huang[1], Miaomiao Chen[1], Yujie Gou[1], Yu Xue[1,2]*, Luoying Zhang[1,3]*

[1]Key Laboratory of Molecular Biophysics of Ministry of Education, Hubei Bioinformatics and Molecular Imaging Key Laboratory, College of Life Science and Technology, Huazhong University of Science and Technology, Wuhan, Hubei 430074, China

[2]Nanjing University Institute of Artificial Intelligence Biomedicine, Nanjing, Jiangsu 210031, China

[3]Hubei Province Key Laboratory of Oral and Maxillofacial Development and Regeneration, Wuhan, Hubei 430022, China

[†]These authors contributed equally to this work.

*To whom correspondence should be addressed.

Luoying Zhang, E-mail: zhangluoying@hust.edu.cn.

Yu Xue, E-mail: xueyu@hust.edu.cn.

Supplementary Figures

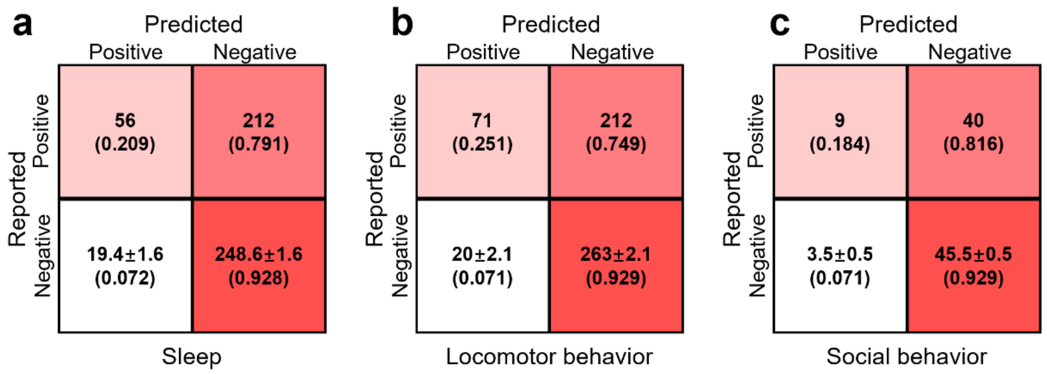

Supplementary Fig. 1: Confusion matrices for assessing the performance of LLM interpretations regarding sleep, locomotor and social activities in fruit flies. The standard error of the mean (SEM) values were calculated for false positive and true negative hits.

a Confusion matrix of LLM interpretation for sleep in fruit flies.

b Confusion matrix of LLM interpretation for locomotor activity in fruit flies.

c Confusion matrix of LLM interpretation for social activity in fruit flies.

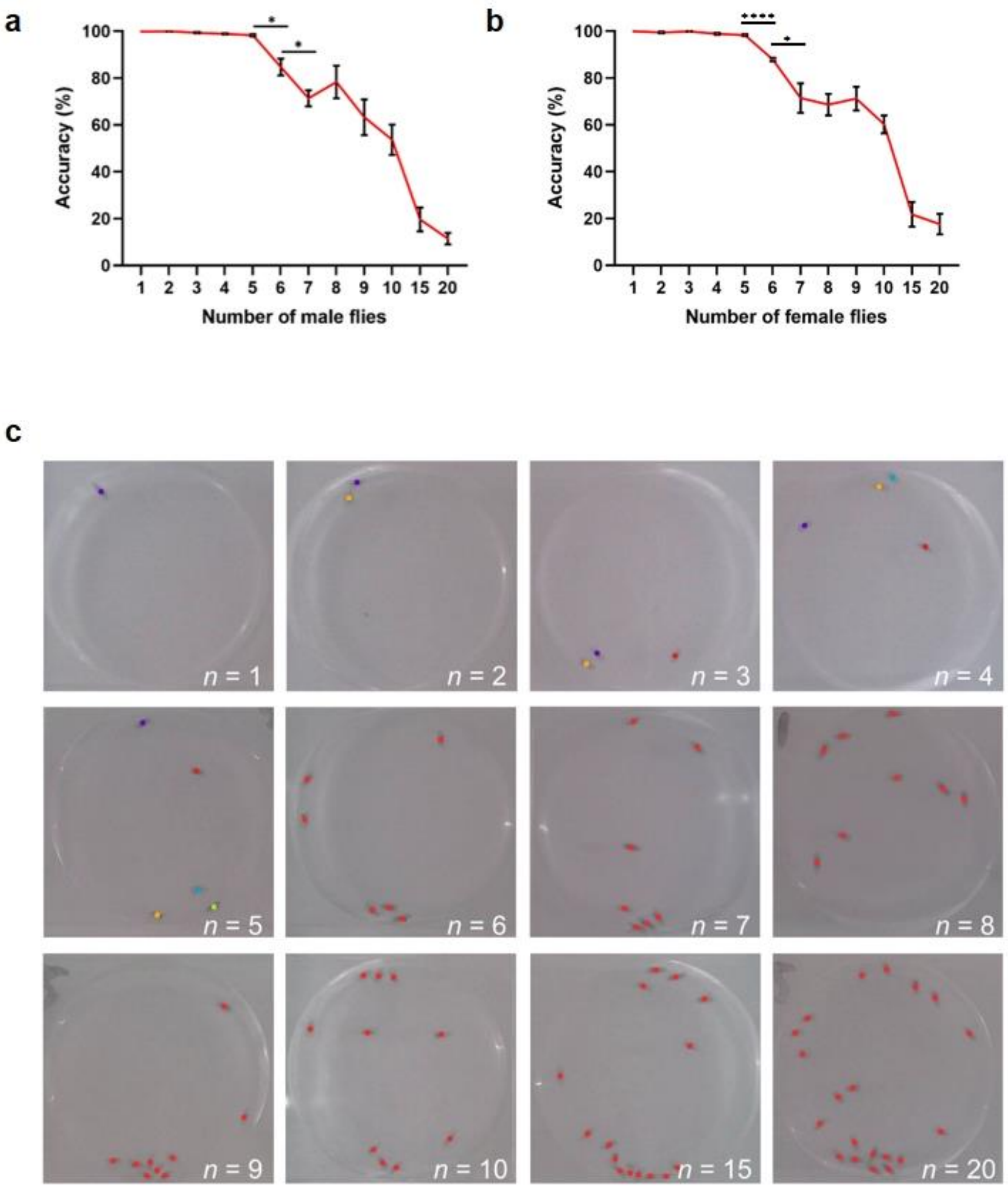

Supplementary Fig. 2: Measurement of the accuracy of movement tracking in fly groups of varying sizes.

a, b Different number of **(a)** male and **(b)** female flies ranging from 1 to 20 were separately recorded in the culture plate, and the accuracy was calculated by manually analyzing 153,360 frames derived from recording videos. The statistical significance of the differences was calculated using the Welch's *t* test. (*n* = 6 independent video fragments, one-sided Welch's *t* test for unpaired comparisons: for male fly, group size 5 *vs.* 6, * p = 0.01249, group size 6 *vs.* 7,

* $p = 0.02333$; for female fly, group size 5 *vs.* 6, **** $p = 0.0000004861$, group size 6 *vs.* 7, * $p = 0.04715$). Data are presented as the mean ± SEM. Source data are provided as a Source Data file.

c Representative images of video recording of flies in group sizes ranging from 1 to 20.

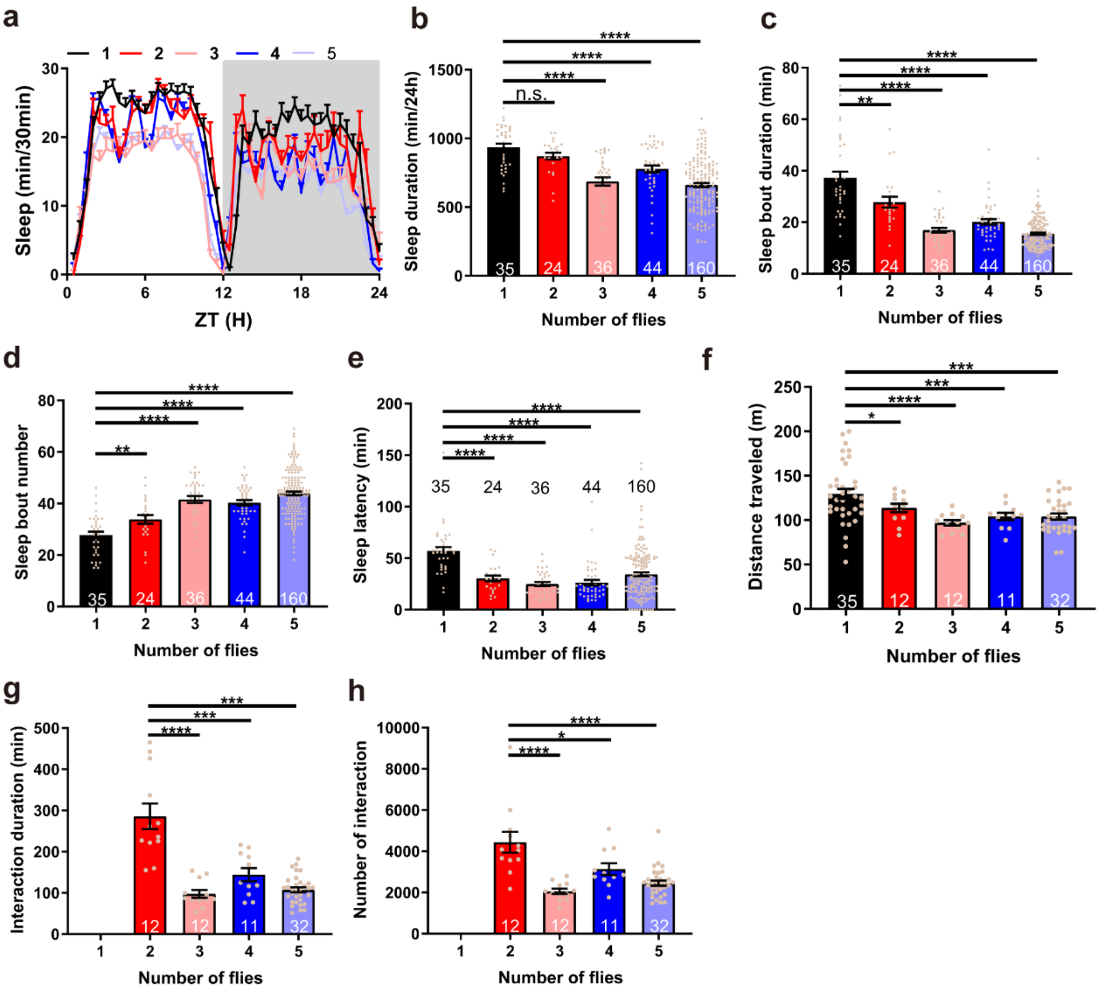

Supplementary Fig. 3: Sleep, locomotor and social activity of isolated fly and flies in groups of various sizes. Male flies are monitored under 12 h light/12 h dark (12L12D) condition.

a Sleep profile of wild-type *w*^{1118} flies in isolation or groups of various sizes ranging from 2 to 5. Gray shade indicates the dark period. The number of flies tested is denoted in (**b**).

b-e Daily sleep duration **(b)** (*****p* < 0.0001, n.s., not significant), sleep bout duration (**c**) (***p* = 0.0035, *****p* < 0.0001), sleep bout number (**d**) (***p* = 0.0056, *****p* < 0.0001) and sleep latency (**e**) (*****p* < 0.0001) of wild-type *w*^{1118} flies in isolation or groups of various sizes ranging from 2 to 5.

f-h Daily distance travelled **(f)** (from left to right, **p* = 0.0389, *****p* < 0.0001, ****p* = 0.0008, ****p* = 0.0003), social interaction duration **(g)** (from left to right, *****p* < 0.0001, ****p* = 0.0009, ****p* = 0.0001) and number of interactions **(h)** (**p* =

0.0317, ****$p < 0.0001$) of wild-type w^{1118} flies in isolation or groups of various sizes ranging from 2 to 5.

The number of flies (**b-e**)/wells (**f-h**) tested is denoted on or above each bar. Two-tailed Mann-Whitney test was used in (**b, c, e, h**). Two-tailed Welch's *t* test was used in (**d, f, g**). Data are presented as the mean ± SEM. Source data are provided as a Source Data file.

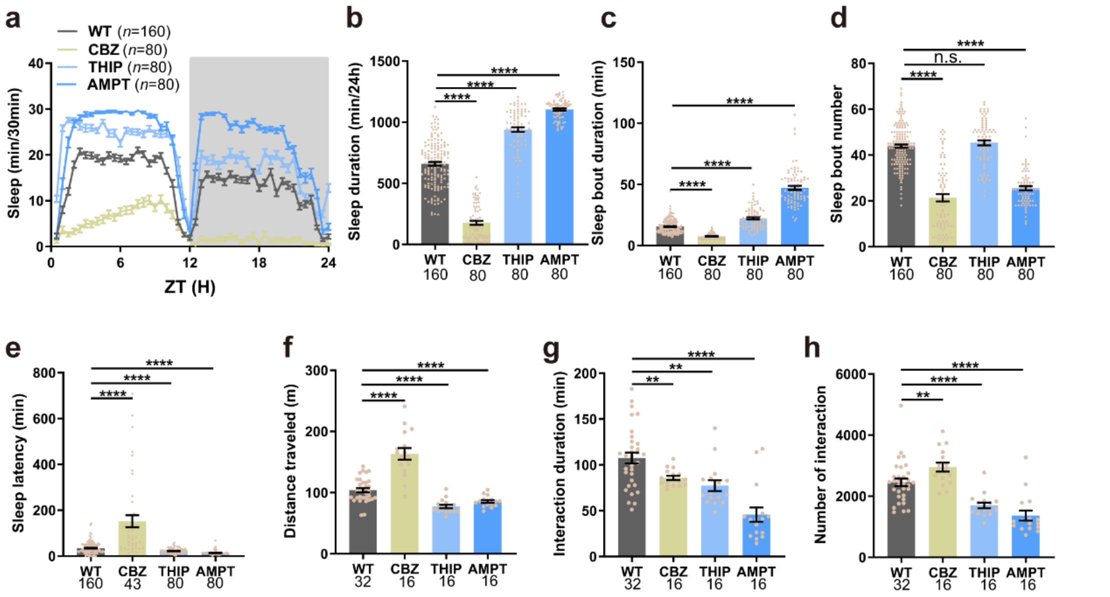

Supplementary Fig. 4: Validation of the video tracking system using drugs that are known to alter sleep, locomotor and social activity. Male flies are monitored under 12L12D condition.

a Sleep profile of wild-type *w1118* flies fed with different drugs and controls in groups of 5. Gray shade indicates the dark period. The number of flies tested is indicated in brackets.

b-e Daily sleep duration **(b)**, sleep bout duration (**c**), sleep bout number (**d**) and sleep latency (**e**) of wild-type w^{1118} flies fed with different drugs and controls in groups of 5. Two-tailed Mann-Whitney test, ****p < 0.0001, n.s., not significant.

f-h Daily distance travelled **(f)** (Two-tailed Welch's t test, ****p < 0.0001), social interaction duration **(g)** (Two-tailed Welch's t test, control vs. CBZ **p = 0.0079; two-tailed Mann-Whitney test, control vs. THIP **p = 0.0017, control vs. AMPT ****p < 0.0001) and number of interactions **(h)** (two-tailed Mann-Whitney test, **p = 0.0079, ****p < 0.0001) of wild-type w^{1118} flies fed with different drugs and controls in groups of 5.

The number of flies (**b-e**)/wells (**f-h**) tested is denoted below each bar. Data are presented as the mean ± SEM. Source data are provided as a Source Data file.

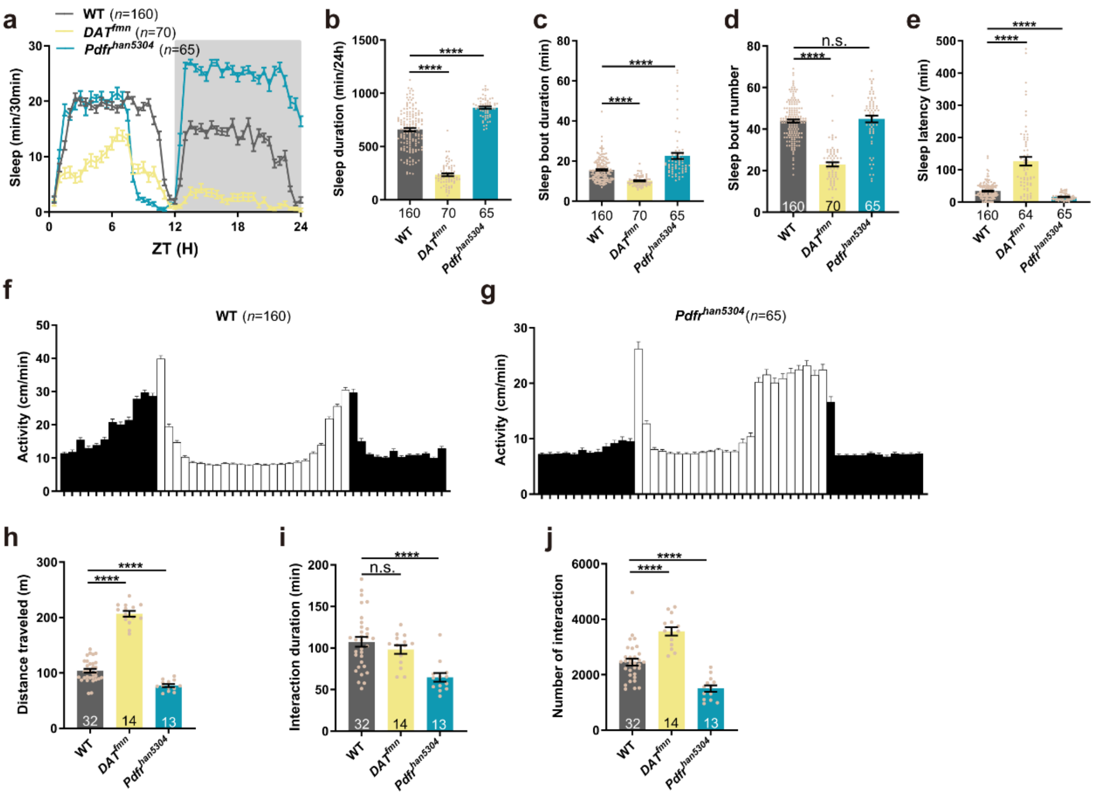

Supplementary Fig. 5: Validation of the video tracking system using mutants that are known to alter sleep, locomotor and social activity. Male flies are monitored under 12L12D condition.

a Sleep profile of mutant flies and wild-type w^{1118} controls in groups of 5. Gray shade indicates the dark period. The number of flies tested is indicated in brackets.

b-e Daily sleep duration **(b)**, sleep bout duration (**c**), sleep bout number (**d**) and sleep latency (**e**) of mutant flies and controls in groups of 5. Two-tailed Mann-Whitney test, ****p < 0.0001.

f, g Activity profile of mutant flies and controls in groups of 5. Black bars represent night and white bars represent day.

h-j Daily distance travelled (**h**), social interaction duration (**i**) and number of interactions (**j**) of mutants and controls. Two-tailed Mann-Whitney test, ****p < 0.0001.

The number of flies (**b-e**)/wells (**h-j**) tested is denoted on or below each bar. Data are presented as the mean ± SEM. Source data are provided as a Source

Data file.

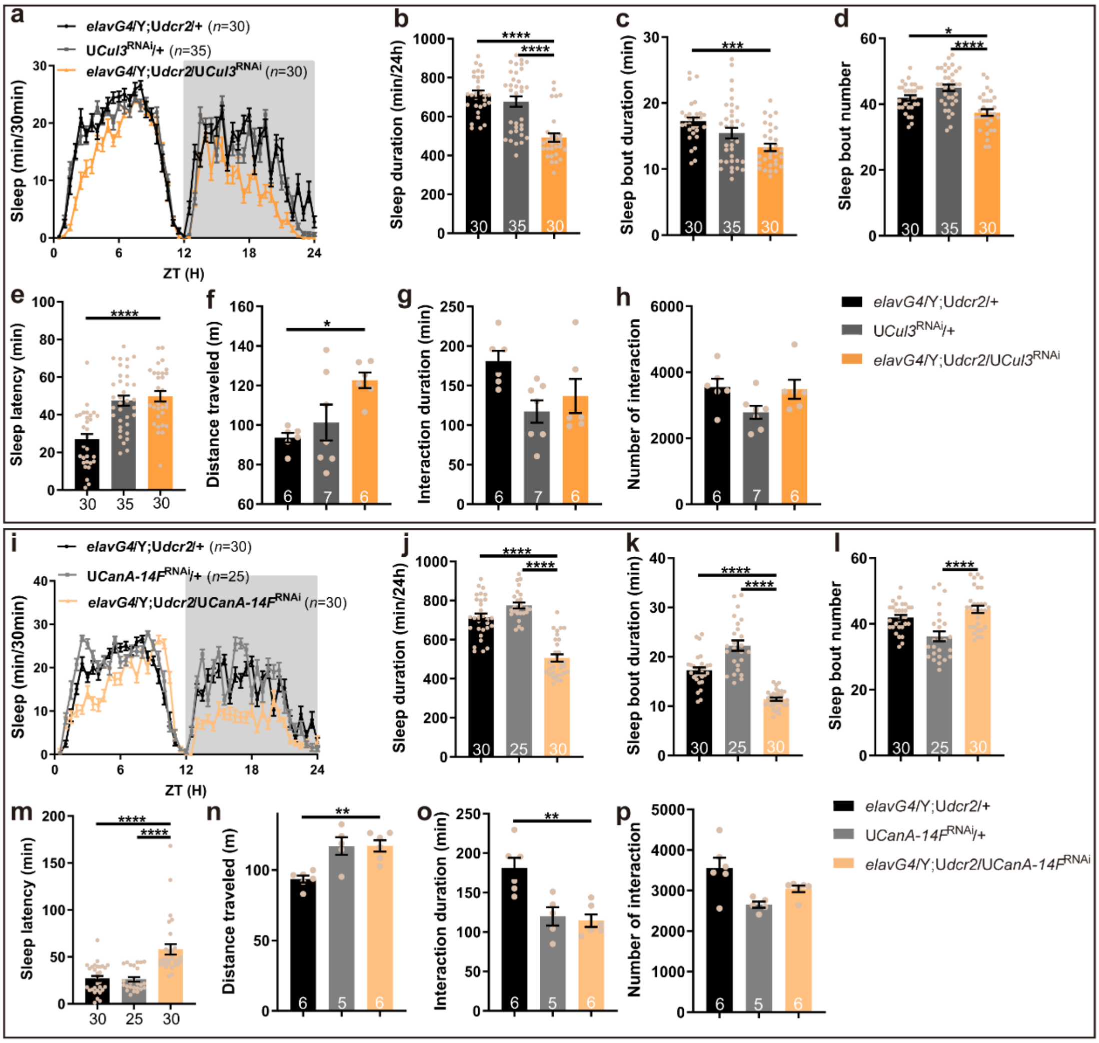

Supplementary Fig. 6: Validation of the video tracking system by knocking down known sleep-regulating genes. Male flies are monitored under 12L12D condition.

a Sleep profile of flies with pan-neuronal knock-down of *Cul3* and controls in groups of 5. Gray shade indicates the dark period. The number of flies tested is indicated in brackets.

b-h Daily sleep duration (**b**) (**** $p < 0.0001$), sleep bout duration (**c**) (***p = 0.0003), sleep bout number (**d**) (**p = 0.0031, ****$p < 0.0001$), sleep latency (**e**) (****$p < 0.0001$), distance travelled (**f**) (*p = 0.0129), social interaction duration (**g**) and number of interactions (**h**) of flies with pan-neuronal knock-down of *Cul3* and controls in groups of 5.

i Sleep profile of flies with pan-neuronal knock-down of *CanA-14F* and controls

in groups of 5. Gray shade indicates the dark period. The number of flies tested is indicated in brackets.

j-p Daily sleep duration (**j**) (*****p* < 0.0001), sleep bout duration (**k**) (*****p* < 0.0001), sleep bout number (**l**) (*****p* < 0.0001), sleep latency (**m**) (*****p* < 0.0001), distance travelled (**n**) (***p* = 0.0023), social interaction duration (**o**) (***p* = 0.0012) and number of interactions (**p**) of flies with pan-neuronal knock-down of *CanA-14F* and controls in groups of 5.

The number of flies (**b-e, j-m**)/wells (**f-h, n-p**) tested is denoted on or below each bar. Kruskal-Wallis and Dunn's multiple comparisons test was used in (**b, g, h, j, l, m, p**). One-way ANOVA and Sidak's multiple comparisons test was used in (**c, d, e, f, k, n, o**). Data are presented as the mean ± SEM. G4, GAL4; U, UAS. Source data are provided as a Source Data file.

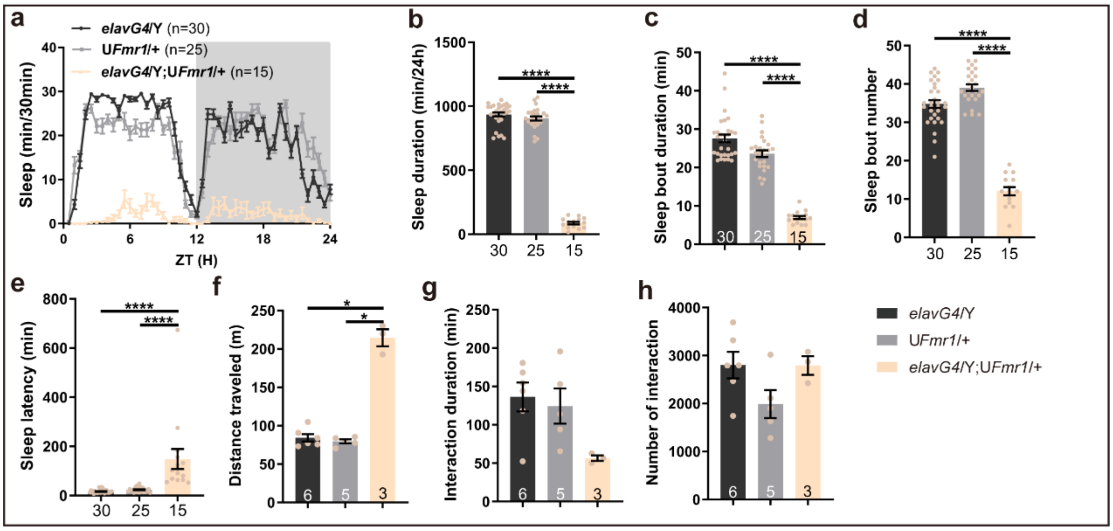

Supplementary Fig. 7: Validation of the video tracking system by overexpressing known sleep-regulating gene. Male flies are monitored under 12L12D condition.

a Sleep profile of flies with pan-neuronal over-expression of *Fmr1* and controls in groups of 5. Gray shade indicates the dark period. The number of flies tested is indicated in brackets.

b-h Daily sleep duration (**b**) (*****p* < 0.0001), sleep bout duration (**c**) (*****p* < 0.0001), sleep bout number (**d**) (*****p* < 0.0001), sleep latency (**e**) (*****p* < 0.0001), distance travelled (**f**) (from left to right, **p* = 0.0484, **p* = 0.0309), social interaction duration (**g**) and number of interactions (**h**) of flies with pan-neuronal over-expression of *Fmr1* and controls in groups of 5.

The number of flies (**b-e**)/wells (**f-h**) tested is denoted on or below each bar. Kruskal-Wallis and Dunn's multiple comparisons test was used in (**b, c, e, f, g, h**). One-way ANOVA and Sidak's multiple comparisons test was used in (**d**). Data are presented as the mean ± SEM. G4, GAL4; U, UAS. Source data are provided as a Source Data file.

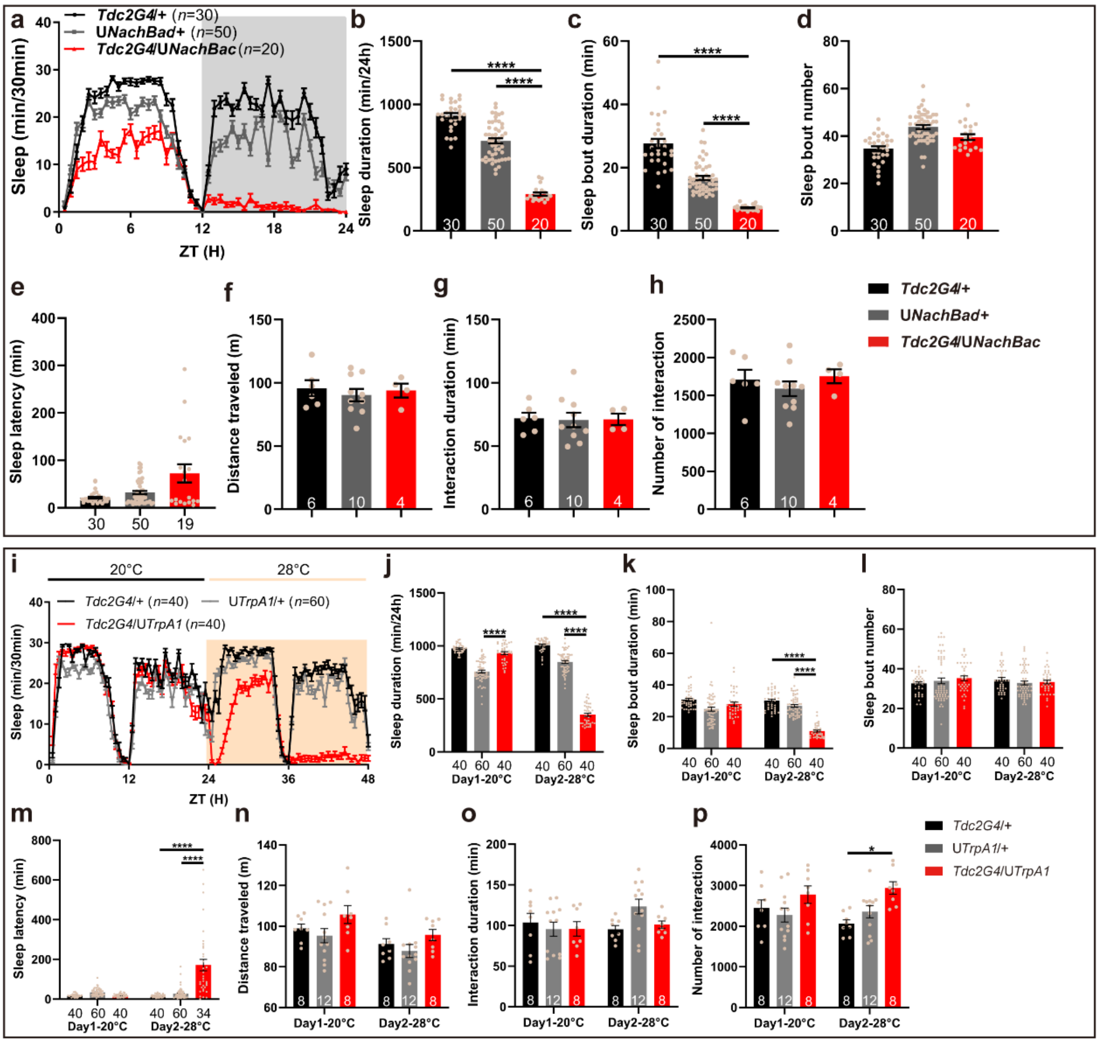

Supplementary Fig. 8: Validation of the video tracking system by activating octopaminergic neurons. Male flies are monitored under 12L12D condition.

a Sleep profile of controls and flies with octopaminergic neurons activated by *NachBac* in groups of 5. Gray shade indicates the dark period. The number of flies tested is indicated in brackets.

b-h Daily sleep duration (**b**) (****$p < 0.0001$), sleep bout duration (**c**) (****$p < 0.0001$), sleep bout number (**d**), sleep latency (**e**), distance travelled (**f**), social interaction duration (**g**) and number of interactions (**h**) of controls and flies with octopaminergic neurons activated by *NachBac* in groups of 5.

i Sleep profile of controls and flies with octopaminergic neurons activated by *TrpA1* in groups of 5. Light orange shade indicates the higher temperature day.

The number of flies tested is indicated in brackets.

j-p Daily sleep duration (**j**) (****p < 0.0001), sleep bout duration (**k**) (****p < 0.0001), sleep bout number (**l**), sleep latency (**m**) (****p < 0.0001), distance travelled (**n**), social interaction duration (**o**) and number of interactions (**p**) (*p = 0.0165) of controls and flies with octopaminergic neurons activated by *TrpA1* in groups of 5.

The number of flies (**b-e, j-m**)/wells (**f-h, n-p**) tested is denoted on or below each bar. Kruskal-Wallis and Dunn's multiple comparisons test was used in (**b, c, e**). One-way ANOVA and Sidak's multiple comparisons test was used in (**d, f, g, h**). Two-way ANOVA and Tukey's multiple comparisons test was used in (**j-p**). Data are presented as the mean ± SEM. G4, GAL4; U, UAS. Source data are provided as a Source Data file.

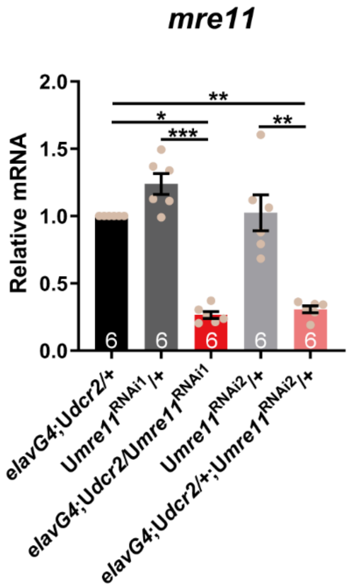

Supplementary Fig. 9: Validation of the RNAi knock-down efficiency for *mre11*.

Relative mRNA level of *mre11* in whole heads of *mre11* RNAi and control flies assessed by quantitative PCR. Kruskal-Wallis and Dunn's multiple comparisons test, **p* = 0.0414, ***p = 0.0006, ***p* = 0.0059, ***p* = 0.0059.

The number of independent experiments tested is denoted on each bar. Data are presented as the mean ± SEM. G4, GAL4; U, UAS. Source data are provided as a Source Data file.

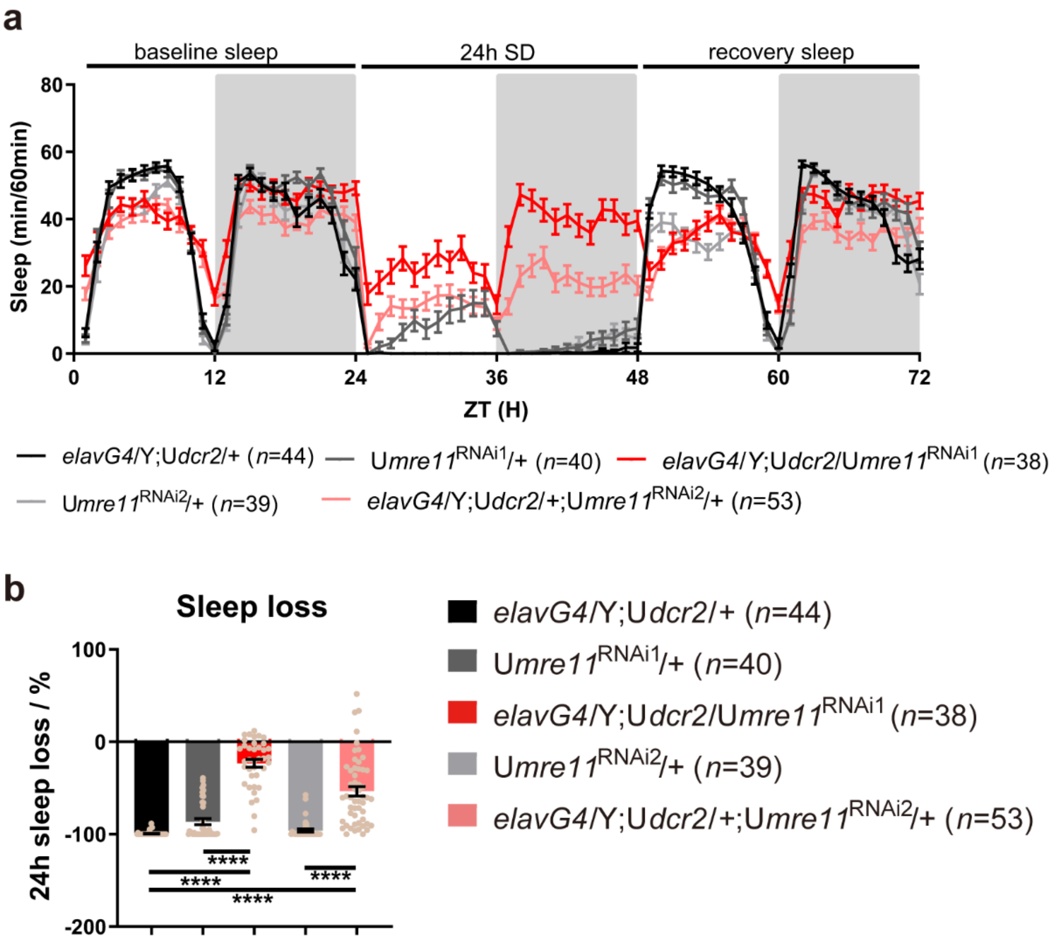

Supplementary Fig. 10: Pan-neuronal knockdown of *mre11* increases resistance to sleep deprivation.

a Sleep profile of *mre11* RNAi and control flies before, during and after sleep deprivation. **b** Sleep loss of *mre11* RNAi and control flies during 24 h sleep deprivation. Kruskal-Wallis and Dunn's multiple comparisons test, $^{****}p < 0.0001$.

The number of independent experiments tested is indicated in brackets. Data are presented as the mean ± SEM. G4, GAL4; U, UAS. Source data are provided as a Source Data file.

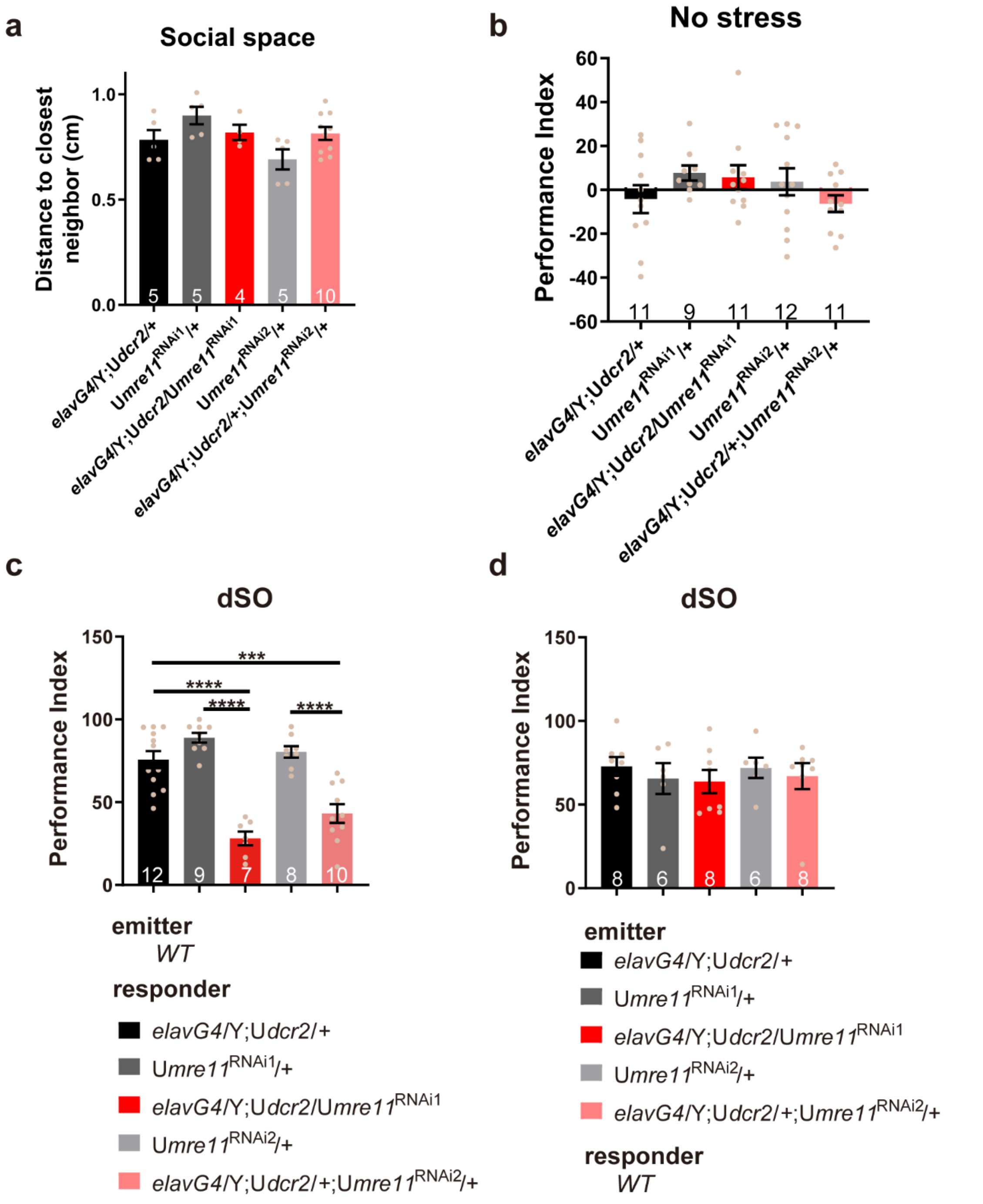

Supplementary Fig. 11: Pan-neuronal knockdown of *mre11* impairs the avoidance of stress odorant but has no effect on social space.

a Distance of each fly to its closet neighbor in the social.

b Performance index of *mre11* RNAi and control flies on the *Drosophila* stress odorant (dSO) test when the emitter flies did not undergo mechanical stress treatment and thus have not emitted stress odorant.

c Performance index of *mre11* RNAi and control flies on the dSO test when when

wild-type *w*^{1118} flies were used as the emitter. The significant differences between *mre11* RNAi and controls reflect impaired response of *mre11* RNAi flies to stress odorant.

d Performance index of wild-type *w*^{1118} flies on the dSO test when *mre11* RNAi and control flies were used as the emitter. No significant difference between *mre11* RNAi and controls indicate that stress odorant emission is intact in *mre11* RNAi flies.

One-way ANOVA and Sidak's multiple comparisons test, $^{***}p = 0.0001$, $^{****}p < 0.0001$.

The number of independent experiments tested is denoted on or below each bar. Data are presented as the mean ± SEM. G4, GAL4; U, UAS. Source data are provided as a Source Data file.

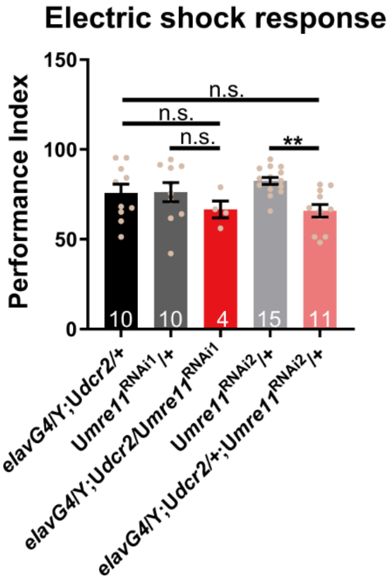

Supplementary Fig. 12: Pan-neuronal knockdown of *mre11* does not affect the avoidance of electric shock.

Performance index of of *mre11* RNAi and control flies in response to electric shock. A higher preference index indicates increased avoidance of electric shock. One-way ANOVA and Sidak's multiple comparisons test, $**p = 0.0016$, n.s., not significant.

The number of independent experiments tested is denoted on each bar. Data are presented as the mean ± SEM. G4, GAL4; U, UAS. Source data are provided as a Source Data file.

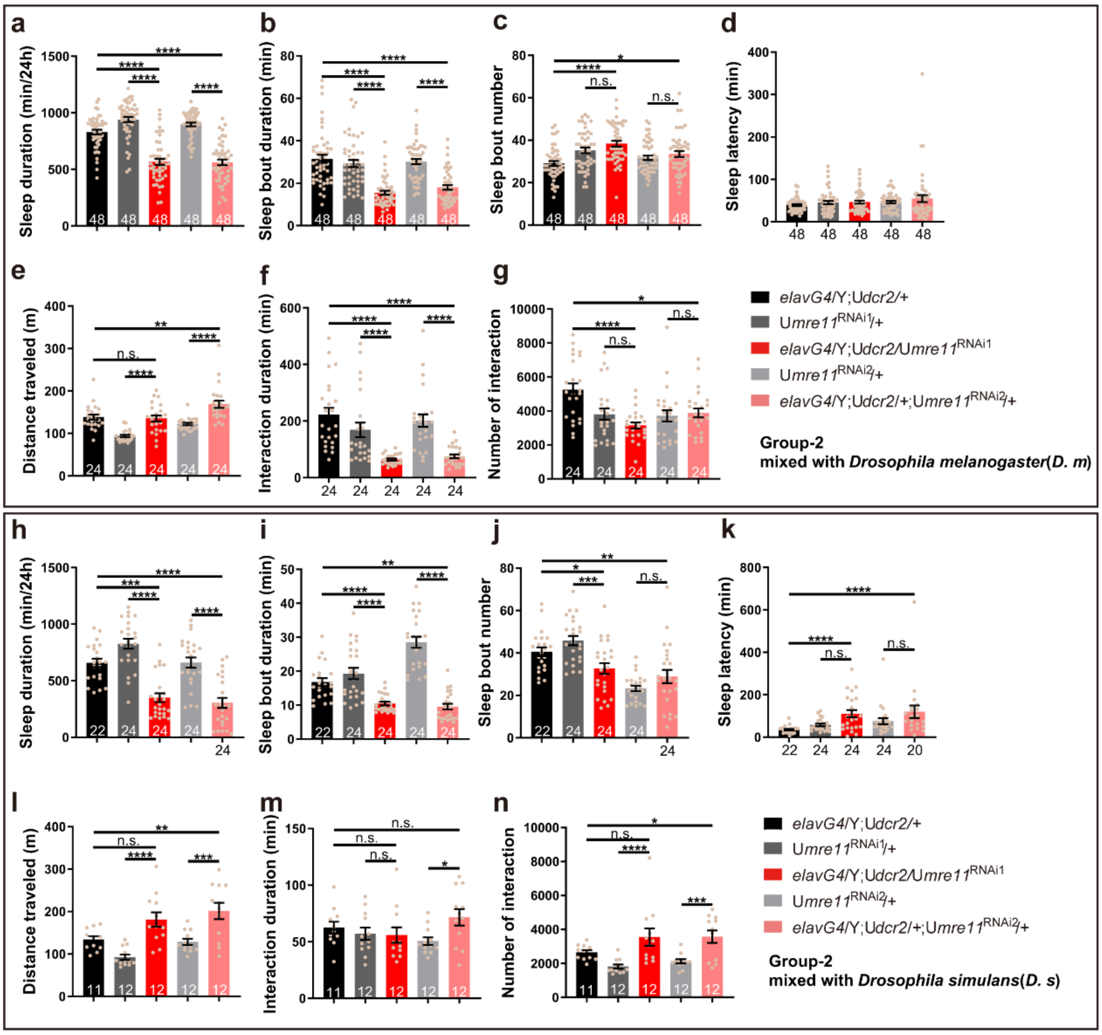

Supplementary Fig. 13: Sleep, locomotor and social activity of *mre11* RNAi flies in the presence of a heterospecific. Male flies are maintained under 12L12D condition.

a-d Daily sleep duration **(a)** (****$p < 0.0001$), sleep bout duration (**b**) (****$p < 0.0001$), sleep bout number (**c**) (****$p < 0.0001$，*$p = 0.0262$, n.s., not significant) and sleep latency (**d**) of a single *mre11* RNAi or control fly with a fly of the w^{1118} strain of *Drosophila melanogaster* (*D. m*).

e-g Daily distance travelled **(e)** (****$p < 0.0001$，**$p = 0.0074$, n.s., not significant), social interaction duration **(f)** (****$p < 0.0001$) and number of interactions **(g)** (****$p < 0.0001$，*$p = 0.0179$, n.s., not significant) of a single *mre11* RNAi or control fly with a fly of the w^{1118} strain of *Drosophila melanogaster* (*D. m*).

h-k Daily sleep duration **(h)** (***$p = 0.0005$, ****$p < 0.0001$), sleep bout duration

(**i**) (**$p = 0.0021$, ****$p < 0.0001$), sleep bout number (**j**) (*$p = 0.0372$, **$p = 0.0013$, ***$p = 0.0002$, n.s., not significant) and sleep latency (**k**) (****$p < 0.0001$, n.s., not significant) of a single *mre11* RNAi or control fly with a fly of the strain w^{501} of *Drosophila simulans* (*D. s*).

l-n Daily distance travelled **(l)** (****$p < 0.0001$, n.s., not significant. **p =0.0020, ***p =0.0007), social interaction duration **(m)** (*p =0.0208, n.s., not significant) and number of interactions **(n)** (****$p < 0.0001$. *p =0.0226, ***p =0.0003, n.s., not significant) of a single *mre11* RNAi or control fly with a fly of the strain w^{501} of *Drosophila simulans* (*D. s*).

The number of flies (**a-d, h-k**)/wells (**e-g, l-n**) tested is denoted on or below each bar. Kruskal-Wallis and Dunn's multiple comparisons test was used in (**a-i, k,** RNAi1 *vs.* UAS/GAL4 controls in **l, m, n**). One-way ANOVA and Sidak's multiple comparisons test was used in (**j,** RNAi2 *vs.* UAS/GAL4 controls in **l, m, n**). Data are presented as the mean ± SEM. G4, GAL4; U, UAS. Source data are provided as a Source Data file.

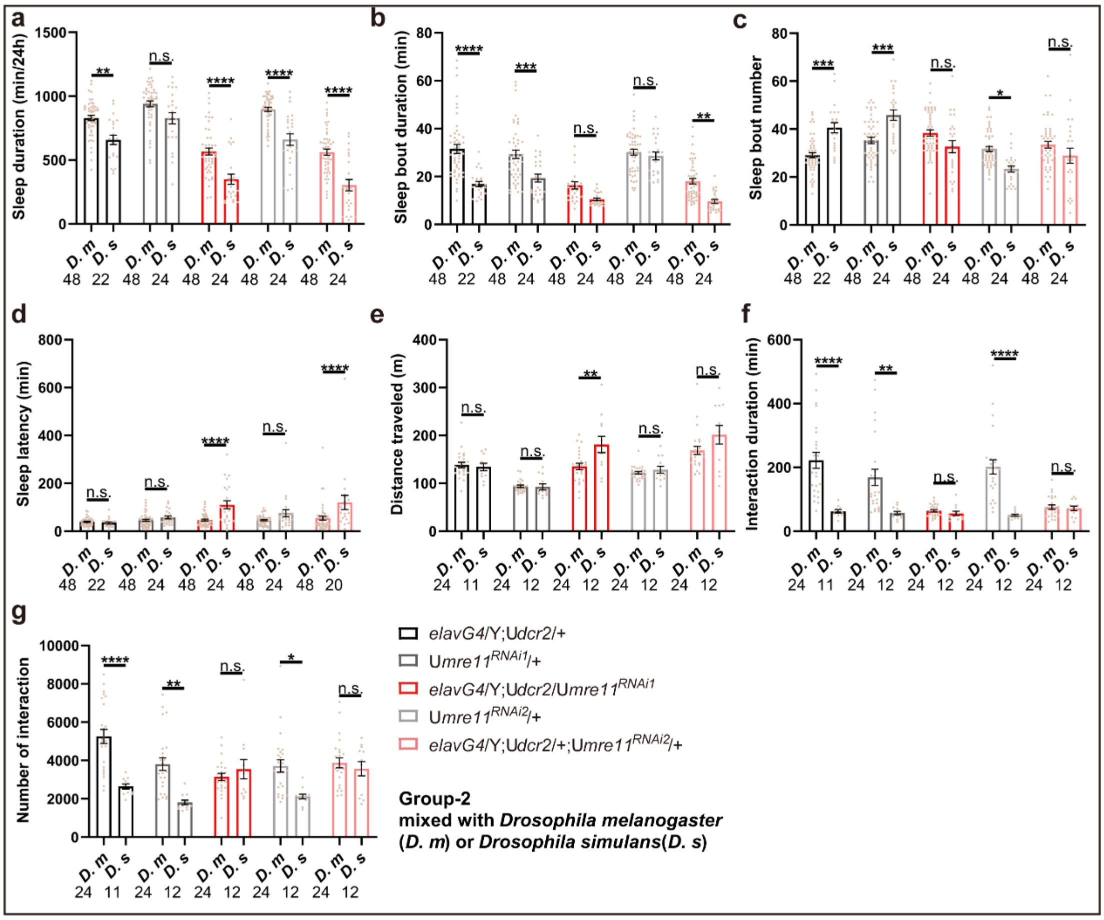

Supplementary Fig. 14: Alteration of sleep, locomotor and social activity of *mre11* RNAi flies in the presence of a heterospecific vs. a conspecific. Male flies are maintained under 12L12D condition.

a-d Daily sleep duration **(a)** (Two-way ANOVA followed by Holm-Sidak's multiple comparisons test; genotype effect $p < 0.0001$, treatment effect $p < 0.0001$, interaction effect $p = 0.1392$, $F_{(4, 348)} = 1.747$; **$p = 0.0026$, ****$p < 0.0001$, n.s., not significant), sleep bout duration (**b**) (Two-way ANOVA followed by Holm-Sidak's multiple comparisons test; genotype effect $p < 0.0001$, treatment effect $p < 0.0001$, interaction effect $p = 0.0016$, $F_{(4, 326)} = 4.450$; ****$p < 0.0001$, ***$p = 0.0003$, **$p = 0.0044$, n.s., not significant), sleep bout number (**c**) (Two-way ANOVA followed by Holm-Sidak's multiple comparisons test; genotype effect $p < 0.0001$, treatment effect $p = 0.5271$, interaction effect $p < 0.0001$, $F_{(4, 348)} = 15.62$; ***$p = 0.0001$, ***$p = 0.0005$, *$p = 0.0151$, n.s., not significant) and sleep latency (**d**) (Two-way ANOVA followed by Holm-Sidak's multiple comparisons test; genotype effect $p < 0.0001$, treatment effect $p < 0.0001$,

interaction effect p=0.0002, $F_{(4, 344)} = 5.683$; ****$p < 0.0001$, n.s., not significant) of a single *mre11* RNAi or control fly with a fly of the strain w^{1118} of *Drosophila melanogaster* (*D. m*) or w^{501} of *Drosophila simulans* (*D. s*).

e-g Daily distance travelled **(e)** (Two-way ANOVA followed by Holm-Sidak's multiple comparisons test; genotype effect $p < 0.0001$, treatment effect $p = 0.0039$, interaction effect $p = 0.0127$, $F_{(4, 169)} = 3.286$; **$p = 0.0064$, n.s., not significant), social interaction duration **(f)** (Two-way ANOVA followed by Holm-Sidak's multiple comparisons test; genotype effect $p < 0.0001$, treatment effect $p < 0.0001$, interaction effect $p < 0.0001$, $F_{(4, 169)} = 7.473$; **$p = 0.0022$, ****$p < 0.0001$, n.s., not significant) and number of interactions **(g)** (Two-way ANOVA followed by Holm-Sidak's multiple comparisons test; genotype effect $p = 0.0027$, treatment effect $p < 0.0001$, interaction effect $p < 0.0001$, $F_{(4, 169)}$ =6.665; ****$p < 0.0001$, **$p = 0.0017$, *$p = 0.0298$, n.s., not significant) of a single *mre11* RNAi or control fly with a fly of the strain w^{1118} of *Drosophila melanogaster* (*D. m*) or w^{501} of *Drosophila simulans* (*D. s*).

The number of flies (**a-d**)/wells (**e-g**) tested is denoted below each bar. Data are presented as the mean ± SEM. G4, GAL4; U, UAS. Source data are provided as a Source Data file.

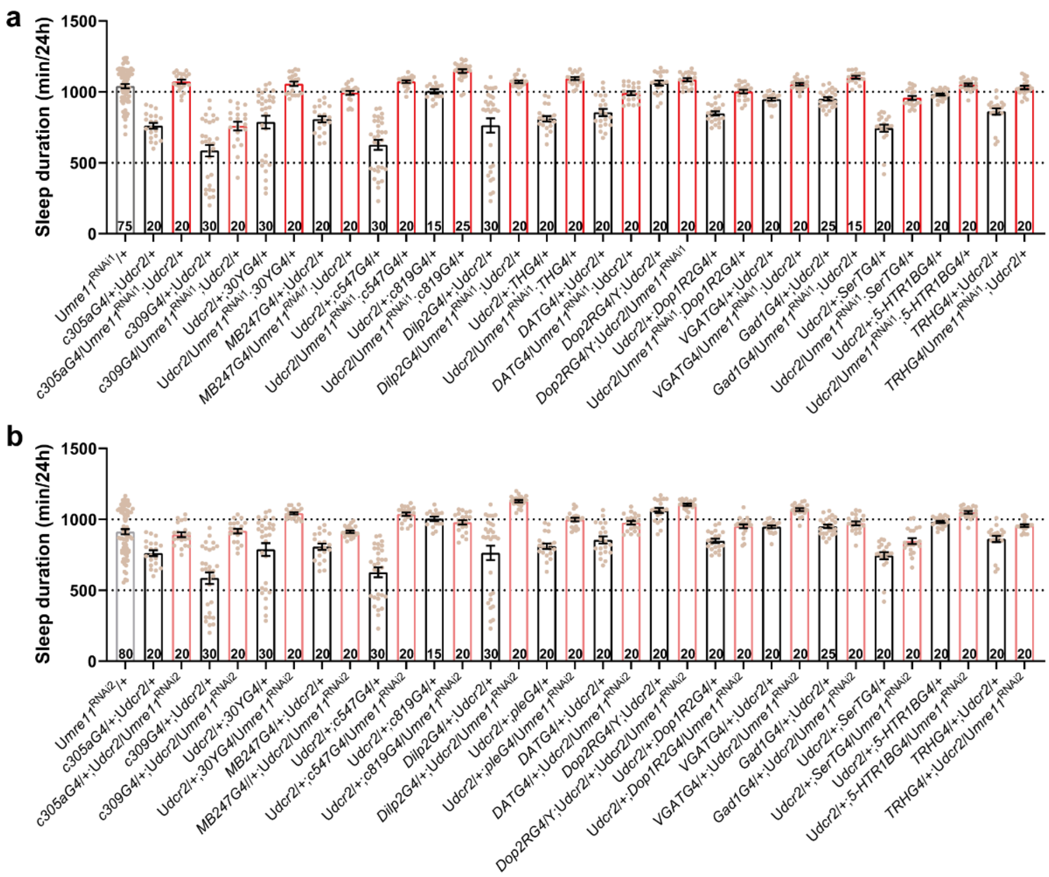

Supplementary Fig. 15: Sleep duration of flies with *mre11* knocked down in different brain structures and cell types.

a, **b** Male flies are maintained under 12L12D condition. Bar graph demonstrates daily sleep duration of flies in group of 5 with *mre11* knocked down using RNAi1 **(a)** and RNAi2 **(b)**.

The number of flies tested is denoted on each bar. Data are presented as the mean ± SEM. G4, GAL4; U, UAS. Source data are provided as a Source Data file.

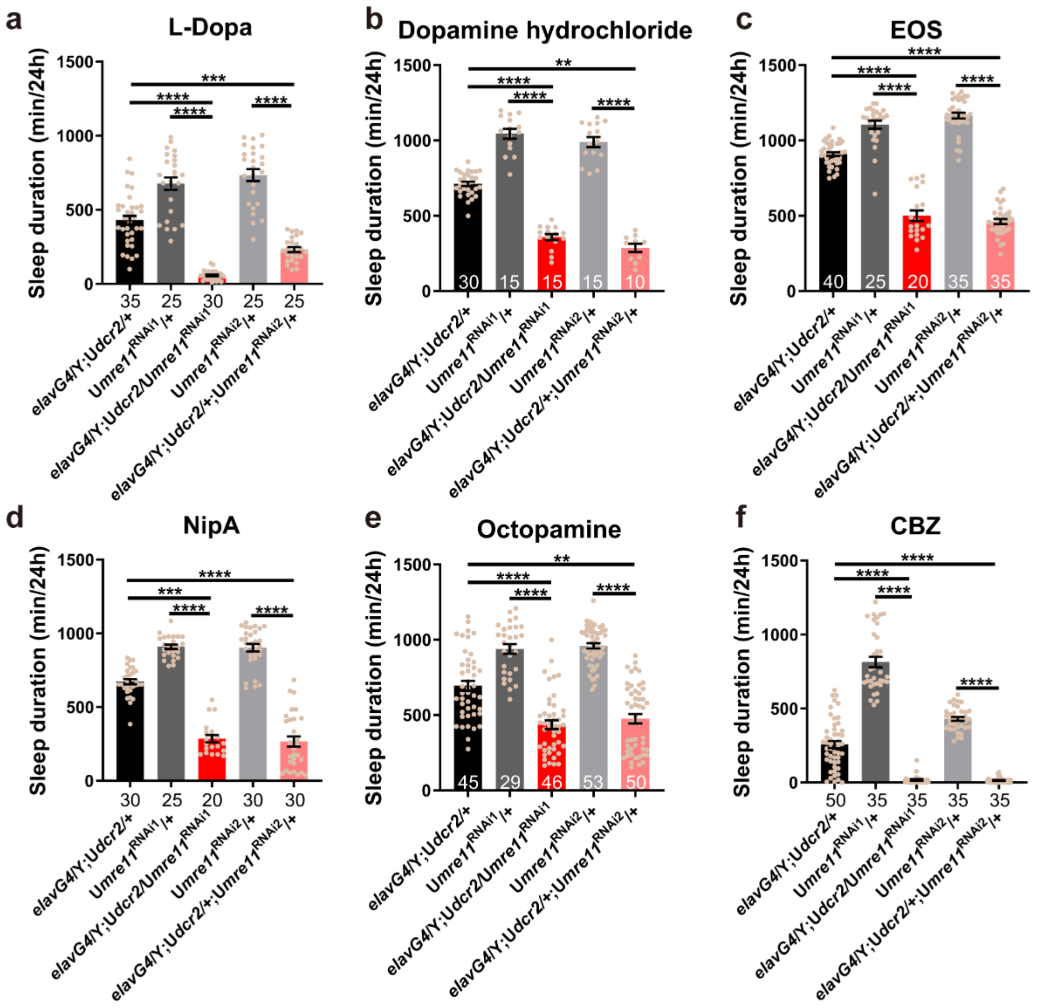

Supplementary Fig. 16: Sleep duration of *mre11* RNAi flies treated with different drugs. Male flies are maintained under 12L12D condition.

a-f Daily sleep duration of mre11 RNAi and control flies in group of 5 treated with different drugs, including L-Dopa **(a)**, dopamine hydrochloride **(b)**, EOS **(c)**, NipA **(d)**, octopamine **(e)** and CBZ **(f)**. In (**a**) and (**b**) one-way ANOVA followed by Sidak's multiple comparison test was used. In (**c-f**) Kruskal-Wallis test followed by Dunn's multiple comparisons test was used, $^{**}p$ = 0.0012, $^{***}p$ = 0.0001, $^{****}p < 0.0001$.

The number of flies tested is denoted on or below each bar. Data are presented as the mean ± SEM. G4, GAL4; U, UAS. Source data are provided as a Source Data file.

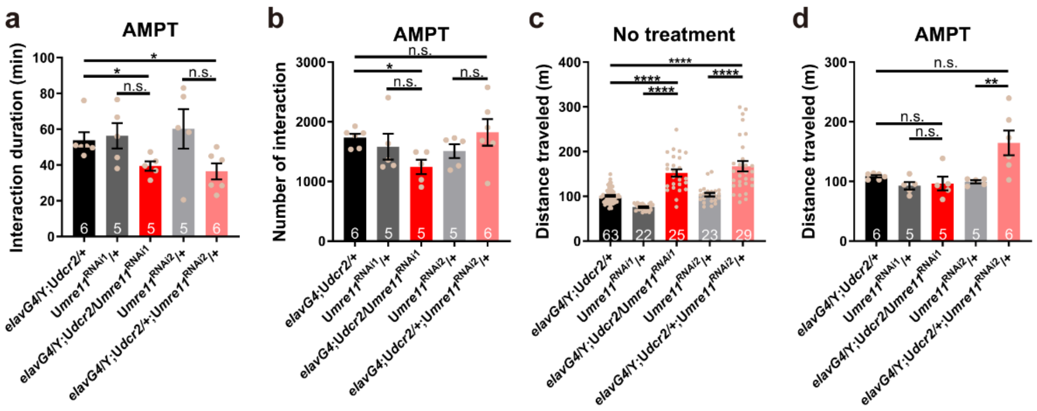

Supplementary Fig. 17: AMPT largely eliminates the influences of *mre11* deficiency on social and locomotor activity. Male flies are maintained under 12L12D condition.

a, b Daily social interaction duration (**a**) (Kruskal-Wallis test and Dunn's multiple comparisons test, from left to right, *p = 0.0415, *p = 0.0320, n.s., not significant) and number of interactions (**b**) (One-way ANOVA and Sidak's multiple comparison test, n.s., not significant) of *mre11* RNAi and control flies when treated with AMPT.

c, d Daily distance travelled by *mre11* RNAi and control flies without (**c**) (Kruskal-Wallis test and Dunn's multiple comparisons test, ****$p < 0.0001$) or with AMPT treatment (**d**) (n = 5-6 wells, One-way ANOVA and Sidak's multiple comparison test, *p = 0.0137, **p = 0.0069, n.s., not significant).

The number of wells tested is denoted on each bar. Data are presented as the mean ± SEM. G4, GAL4; U, UAS. Source data are provided as a Source Data file.

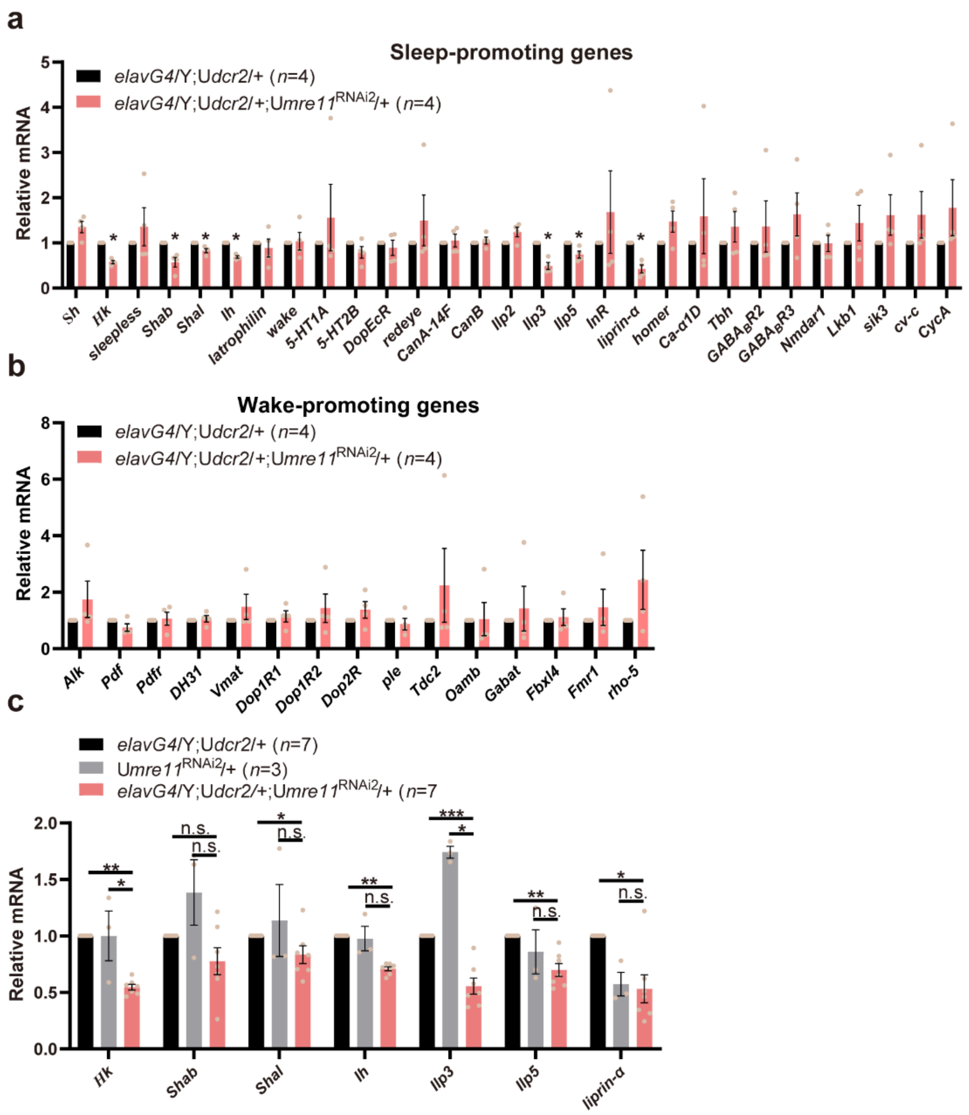

)

Supplementary Fig. 18: Expression of sleep genes in *mre11* RNAi flies.

a, b Relative mRNA level of sleep genes in whole heads of *mre11* RNAi and control flies measured by quantitative PCR. These include sleep-promoting genes (**a**) and wake-promoting genes (**b**). Two-tailed Mann-Whitney test, *$p = 0.0286$.

c Relative mRNA level of differentially expressed sleep genes in (**a**) and (**b**) in *mre11* RNAi and UAS/GAL4 control flies measured by quantitative PCR for further validation. Kruskal-Wallis and Dunn's multiple comparisons test, from left to right, *$p = 0.0491$, **$p = 0.0011$, **$p = 0.0043$, *$p = 0.0170$, n.s., not significant. The number of independent experiments tested is indicated in brackets. Data are presented as the mean ± SEM. G4, GAL4; U, UAS. Source data are provided as a Source Data file.

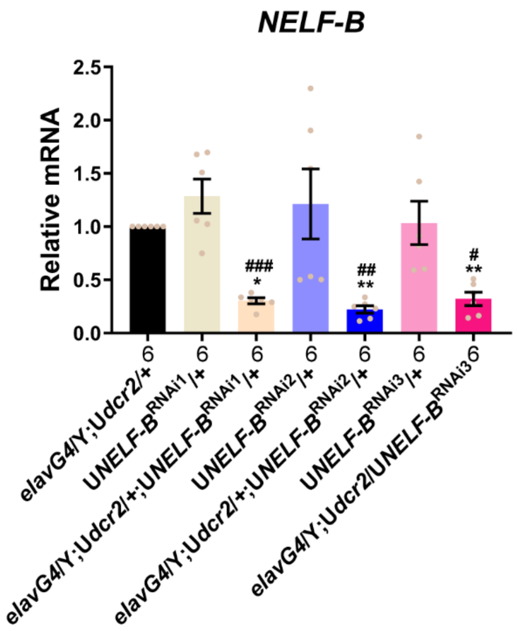

Supplementary Fig. 19: Validation of the RNAi knock-down efficiency for *NELF-B*.

Relative mRNA level of *NELF-B* in whole heads of *NELF-B* RNAi and control flies assessed by quantitative PCR. Kruskal-Wallis and Dunn's multiple comparisons test, from left to right, $^{*}p = 0.0414$, $^{\#\#\#}p = 0.0006$, $^{**/\#\#}p = 0.0059$, $^{**}p = 0.0019$, $^{\#}p = 0.0164$.

The number of independent experiments tested is denoted below each bar. Data are presented as the mean ± SEM. * compared with the GAL4 controls, # compared with the UAS controls. G4, GAL4; U, UAS. Source data are provided as a Source Data file.

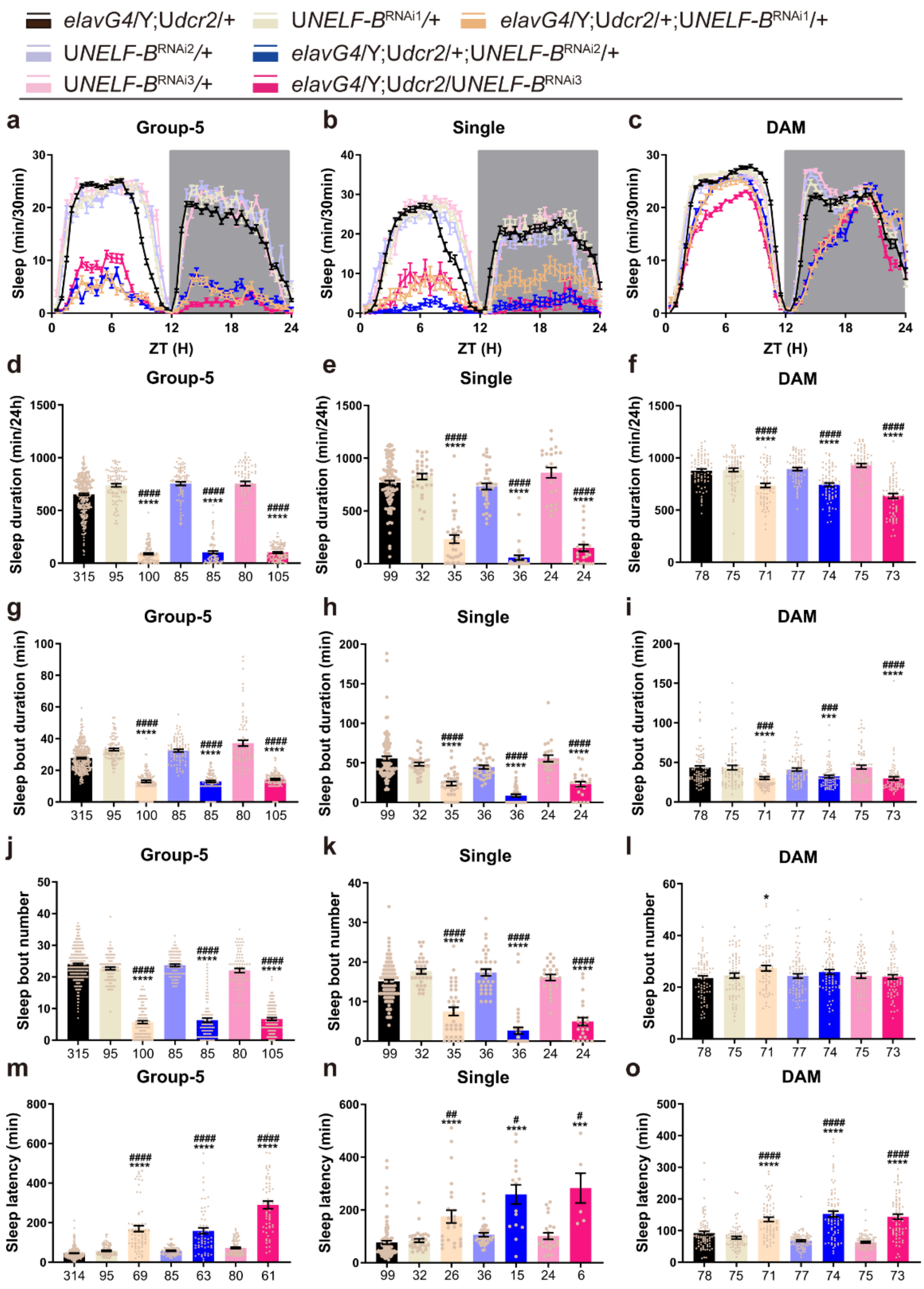

Supplementary Fig. 20: Pan-neuronal knock-down of *NELF-B* reduces sleep under group and isolated condition. Male flies are maintained under 12L12D condition.

a, b, c Sleep profile of *NELF-B* RNAi and control flies in Group-5 (**a**), isolated condition monitored using video recording system (**b**) or DAM (**c**). The number of flies tested is denoted in **d, e** and **f,** respectively.

d, e, f Daily sleep duration of *NELF-B* RNAi and control flies in Group-5 (**d**) ($^{\#\#\#\#/****}p < 0.0001$), isolated condition using video monitoring (**e**) ($^{\#\#\#\#/****}p < 0.0001$) or DAM (**f**) ($n$ = 71-78, One-way ANOVA and Sidak's multiple comparisons test was used to compare RNAi1/RNAi3 flies and UAS/GAL4 controls, $^{\#\#\#\#/****}p < 0.0001$).

g, h, i Sleep bout duration of *NELF-B* RNAi and control flies in Group-5 (**g**) ($^{\#\#\#\#/****}p < 0.0001$), isolated condition using video monitoring (**h**) ($^{\#\#\#\#/****}p < 0.0001$) or DAM (**i**) (from left to right, $^{\#\#\#}p = 0.0001$, $^{\#\#\#}p = 0.0004$, $^{***}p = 0.0002$, $^{\#\#\#\#/****}p < 0.0001$).

j, k, l Sleep bout number of *NELF-B* RNAi and control flies in Group-5 (**j**) ($^{\#\#\#\#/****}p < 0.0001$), isolated condition using video monitoring (**k**) ($^{\#\#\#\#/****}p < 0.0001$) or DAM (**l**) ($^{**}p = 0.0097$).

m, n, o Sleep latency of *NELF-B* RNAi and control flies in Group-5 (**m**) ($^{\#\#\#\#/****}p < 0.0001$), isolated condition using video monitoring (**n**) (from left to right, $^{\#\#}p = 0.0094$, $^{\#}p = 0.0372$, $^{\#}p = 0.0251$, $^{***}p = 0.0001$, $^{****}p < 0.0001$) or DAM (**o**) ($^{\#\#\#\#/****}p < 0.0001$).

The number of flies tested is denoted below each bar. Kruskal-Wallis and Dunn's multiple comparisons test was used in (**d, e, g-k, m-o,** RNAi2 *vs.* UAS/GAL4 controls in **f**). One-way ANOVA and Sidak's multiple comparisons test was used in (**l,** RNAi1/ RNAi3 *vs.* UAS/GAL4 controls in **f**). Data are presented as the mean ± SEM. * compared with the GAL4 controls, # compared with the UAS controls. G4, GAL4; U, UAS. Source data are provided as a Source Data file.

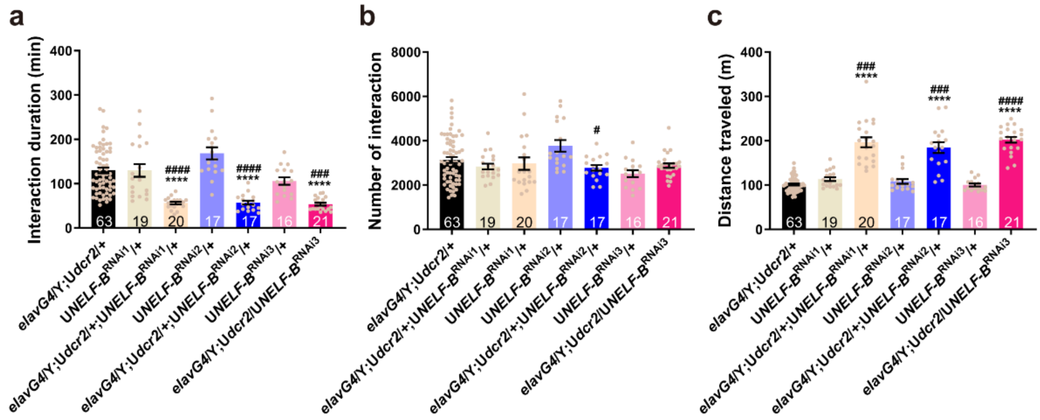

Supplementary Fig. 21: Pan-neuronal knock-down of *NELF-B* reduces social activity and increases locomotor activity. Male flies are maintained under 12L12D condition.

a, b, c Daily social interaction duration (**a**) ($^{\#\#\#}p = 0.0003$, $^{\#\#\#\#/****}p < 0.0001$), number of interactions (**b**) ($^{\#}p = 0.0149$) and distance travelled (**c**) ($^{\#\#\#}p = 0.0001$, $^{\#\#\#\#/****}p < 0.0001$) for *NELF-B* RNAi and control flies in Group-5.

The number of flies tested is denoted on each bar. Kruskal-Wallis and Dunn's multiple comparisons test was used in (**a-c**). Data are presented as the mean ± SEM. * compared with the GAL4 controls, # compared with the UAS controls. G4, GAL4; U, UAS. Source data are provided as a Source Data file.

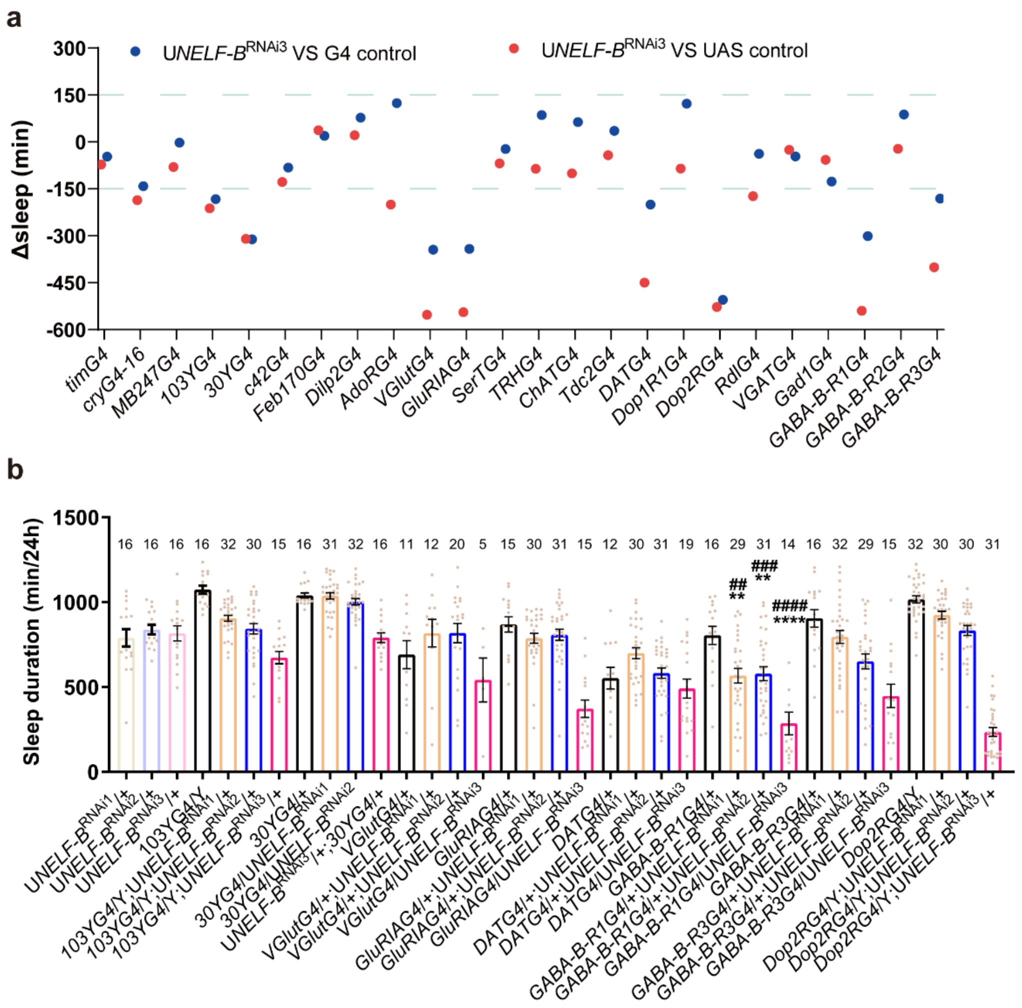

Supplementary Fig. 22: Knocking down *NELF-B* in GABA-B-R1+ cells reduces sleep duration. Male flies are maintained under 12L12D condition and monitored in DAM.

a Alteration of daily sleep duration in *NELF-B* RNAi3 flies compared to UAS (blue dot) and GAL4 (red dot) controls. A positive value indicates that the sleep duration in the RNAi group is longer than that of control, whereas a negative value indicates that the sleep duration in the RNAi group is shorter than that of the control. Raw data and number of flies tested are shown in Source Data.

b Daily sleep duration of *NELF-B* RNAi and control flies using different GAL4 drivers. The number of flies tested is denoted above each bar. One-way ANOVA and Sidak's multiple comparisons test was used to compare RNAi1/RNAi2 flies and UAS/GABA-B-R1GAL4 controls, from left to right, $^{**}p = 0.0020$, $^{\#\#}p = 0.0039$, $^{**}p = 0.0011$, $^{\#\#\#}p = 0.0002$; Kruskal-Wallis and Dunn's multiple comparisons

test was used to compare RNA3 flies and UAS/GABA-B-R1GAL4 controls, $^{###}p = 0.0002$, $^{****}p < 0.0001$.

Data are presented as the mean ± SEM. * compared with the GAL4 controls, # compared with the UAS controls. G4, GAL4; U, UAS. Source data are provided as a Source Data file.

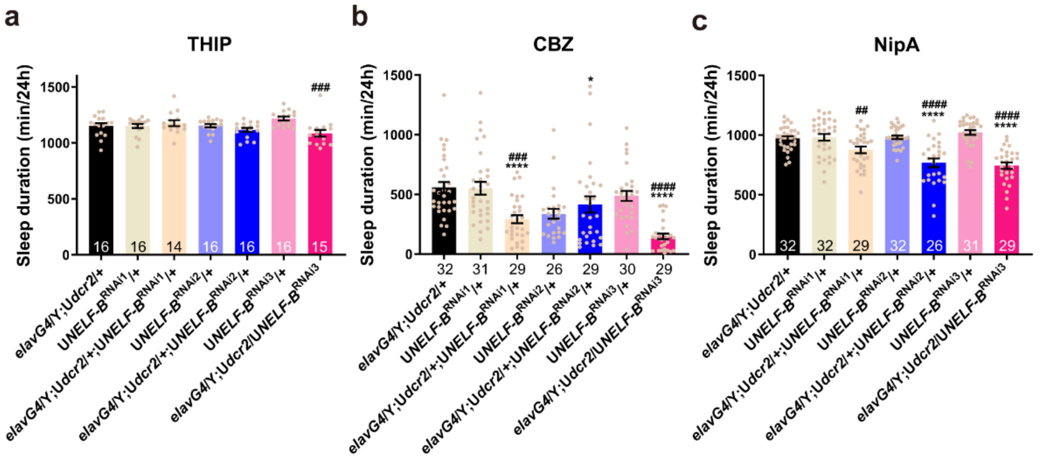

Supplementary Fig. 23: Drugs that influence GABA signaling inhibit or partially inhibit the short-sleep phenotype of *NELF-B* RNAi flies. Male flies are maintained under 12L12D condition and monitored in DAM.

a, b, c Daily sleep duration of *NELF-B* RNAi and control flies treated with different drugs, including THIP (**a**) (One-way ANOVA and Sidak's multiple comparisons test was used to compare RNAi2 flies and UAS/GAL4 controls, Kruskal-Wallis and Dunn's multiple comparisons test was used to compare RNAi1/RNAi3 flies and UAS/GAL4 controls, $^{\#\#\#}p = 0.0002$), CBZ (**b**) (Kruskal-Wallis and Dunn's multiple comparisons test, $^{*}p = 0.0105$, $^{\#\#\#}p = 0.0004$, $^{\#\#\#\#/****}p < 0.0001$) and NipA (**c**) (One-way ANOVA and Sidak's multiple comparisons test was used to compare RNAi1/RNAi2 flies and UAS/GAL4 controls, Kruskal-Wallis and Dunn's multiple comparisons test was used to compare RNAi3 flies and UAS/GAL4 controls, $^{*}p = 0.0163$, $^{\#\#}p = 0.0075$, $^{\#\#\#\#/****}p < 0.0001$).

The number of flies tested is denoted on or below each bar. Data are presented as the mean ± SEM. * compared with the GAL4 controls, # compared with the UAS controls. G4, GAL4; U, UAS. Source data are provided as a Source Data file.

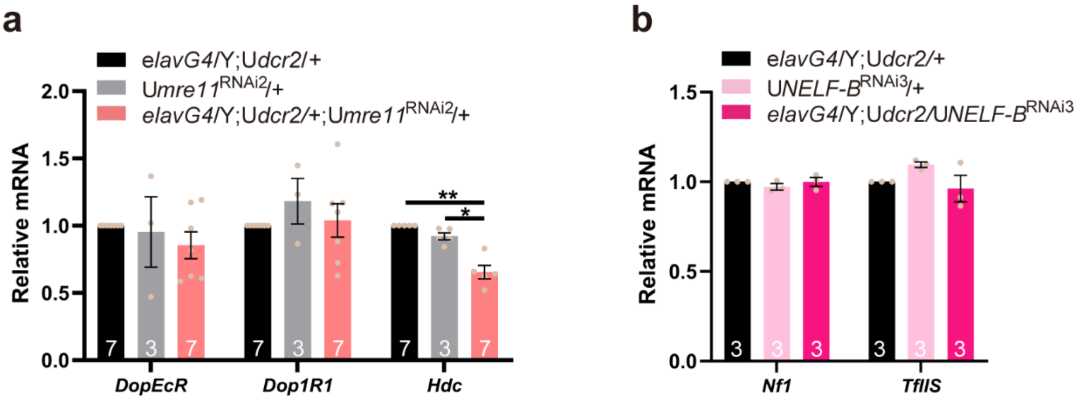

Supplementary Fig. 24: mRNA level of genes predicted to be targets of *mre11* or *NELF-B*.

a Relative mRNA level of genes predicted to be targets of *mre11* in whole heads of *mre11* RNAi and control flies measured by quantitative PCR.

b Relative mRNA level of genes predicted to be targets of *NELF-B* in whole heads of *NELF-B* RNAi and control flies measured by quantitative PCR.

The number of independent experiments tested is denoted on each bar. Kruskal-Wallis and Dunn's multiple comparisons test, **p* =0.0226, ***p* =0.0023. Data are presented as the mean ± SEM. G4, GAL4; U, UAS. Source data are provided as a Source Data file.

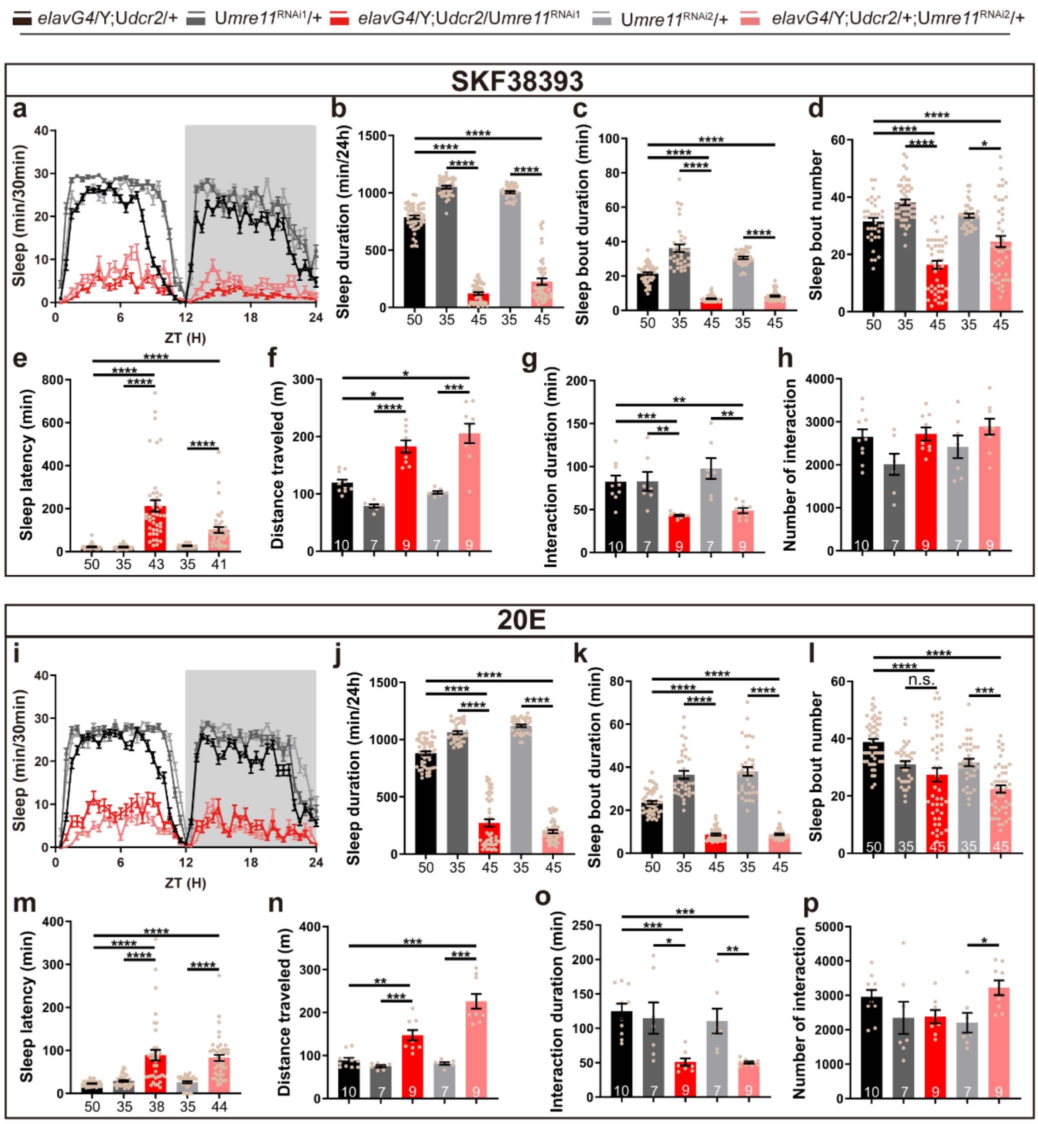

Supplementary Fig. 25: Sleep, locomotor and social activity of *mre11* RNAi flies treated with drugs that target Dop1R and DopEcR. Male flies are maintained under 12L12D condition.

a Sleep profile of *mre11* RNAi and control flies fed with Dop1R agonist SKF38393. Gray shade indicates the dark period. The number of flies tested is denoted in (**b**).

b-e Daily sleep duration **(b)** (****$p < 0.0001$), sleep bout duration (**c**) (****$p < 0.0001$), sleep bout number (**d**) (*$p = 0.0219$, ****$p < 0.0001$) and sleep latency (**e**) (****$p < 0.0001$) of *mre11* RNAi and control flies fed with SKF38393.

f-h Daily distance travelled **(f)** (****$p < 0.0001$), social interaction duration **(g)** (from left to right, ***$p = 0.0010$, **$p = 0.0021$, **$p = 0.0072$, ***$p = 0.0005$) and

number of interactions **(h)** (*$p = 0.0366$) of *mre11* RNAi and control flies fed with SKF38393.

i Sleep profile of *mre11* RNAi and control flies fed with DopEcR ligand 20E. Gray shade indicates the dark period. The number of flies tested is denoted in (**j**).

j-m Daily sleep duration **(j)** (****$p < 0.0001$), sleep bout duration (**k**) (****$p < 0.0001$), sleep bout number (**l**) (****$p < 0.0001$, n.s., not significant) and sleep latency (**m**) (****$p < 0.0001$) of *mre11* RNAi and control flies fed with 20E.

n-p Daily distance travelled **(n)** (from left to right, **$p = 0.0072$, ***$p = 0.0003$, ***$p = 0.0007$, ***$p = 0.0009$), social interaction duration **(o)** (from left to right, **$p = 0.0015$, *$p = 0.0100$, ***$p = 0.0001$, **$p = 0.0028$) and number of interactions **(p)** (*$p = 0.0131$) of *mre11* RNAi and control flies fed with 20E.

The number of flies (**b-e, j-m**)/wells (**f-h, n-p**) tested is denoted on or below each bar. Kruskal-Wallis and Dunn's multiple comparisons test was used in (**b-e, j, k, m, n**). One-way ANOVA and Sidak's multiple comparisons test was used in (**f-h, l, o, p**). Data are presented as the mean ± SEM. G4, GAL4; U, UAS. Source data are provided as a Source Data file.

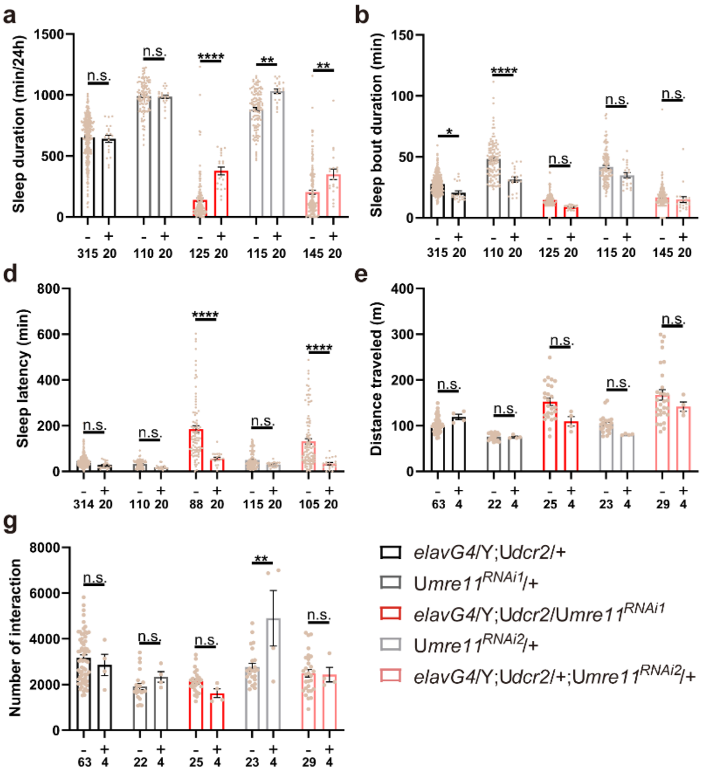

Supplementary Fig. 26: Sleep, locomotor and social activity of *mre11* RNAi flies treated with Dop1R antagonist. Male flies are maintained under 12L12D condition.

a-d Daily sleep duration **(a)** (Two-way ANOVA followed by Holm-Sidak's multiple comparisons test; genotype effect $p < 0.0001$, treatment effect $p < 0.0001$, interaction effect $p < 0.0001$, $F_{(4, 900)} = 7.179$; ****$p < 0.0001$, **$p = 0.0028$, n.s., not significant), sleep bout duration (**b**) (Two-way ANOVA followed by Holm-Sidak's multiple comparisons test; genotype effect $p < 0.0001$, treatment effect $p < 0.0001$, interaction effect $p = 0.0004$, $F_{(4, 900)} = 5.142$; *$p = 0.0298$, ****$p < 0.0001$, n.s., not significant), sleep bout number (**c**) (Two-way ANOVA followed by Holm-Sidak's multiple comparisons test; genotype effect $p < 0.0001$, treatment effect $p < 0.0001$, interaction effect $p < 0.0001$, $F_{(4, 900)} = 48.31$; ****$p < 0.0001$, n.s., not significant) and sleep latency (**d**) (Two-way ANOVA followed by Holm-Sidak's multiple comparisons test; genotype effect $p < 0.0001$, treatment effect $p < 0.0001$, interaction effect $p < 0.0001$, $F_{(4, 882)} = 11.50$; ****$p < 0.0001$, n.s., not significant) of *mre11* RNAi and control flies fed with (+) or without (-) SCH23390.

e-g Daily distance travelled **(e)** (Two-way ANOVA followed by Holm-Sidak's multiple comparisons test; genotype effect $p < 0.0001$, treatment effect $p = 0.0529$, interaction effect $p = 0.0979$, $F_{(4, 172)} = 1.992$; n.s., not significant), social interaction duration **(f)** (Two-way ANOVA followed by Holm-Sidak's multiple comparisons test; genotype effect $p < 0.0001$, treatment effect $p = 0.0102$, interaction effect $p = 0.0026$, $F_{(4, 172)} = 4.247$; ***$p = 0.0005$, n.s., not significant) and number of interactions **(g)** (Two-way ANOVA followed by Holm-Sidak's multiple comparisons test; genotype effect $p < 0.0001$, treatment effect $p = 0.1239$, interaction effect $p = 0.0012$, $F_{(4, 172)} = 4.730$; **$p = 0.0010$, n.s., not significant) of *mre11* RNAi and control flies fed with (+) or without (-) SCH23390.

The number of flies (**a-d**)/wells (**e-g**) tested is denoted below each bar. Data are presented as the mean ± SEM. Source data are provided as a Source Data file.
