# Supplementary material for: Large-language models facilitate discovery of the molecular signatures regulating sleep and activity
Source: Nat Commun. 2024 May 1;15:3685. doi: 10.1038/s41467-024-48005-w (PMC11063160; doi:10.1038/s41467-024-48005-w)
Supplement: Supplementary file 3 — Description of Additional Supplementary Files [file 41467_2024_48005_MOESM3_ESM.pdf]

Description of Additional Supplementary Files

File Name: Supplementary Data 1
Description: The LLM interpretation of gene functions in 3 fly behaviors across the genome.

File Name: Supplementary Data 2
Description: The accuracy of LLM prompt-responses for interpreting genes that function in the 3 behaviors.

File Name: Supplementary Data 3
Description: Experimental design for studying the 3 behaviors using LLMs.

File Name: Supplementary Data 4
Description: Accuracy statistics for tracking varying sizes of fly groups.

File Name: Supplementary Data 5
Description: Strains used in this study.

File Name: Supplementary Data 6
Description: Enrichment analyses of genes that regulate the 3 behaviors.

File Name: Supplementary Data 7
Description: List of qRT-PCR primers used in this study.

File Name: Supplementary Data 8
Description: Constructing a signal web that regulate the 3 behaviors.

File Name: Supplementary Movie 1
Description: Tracking videos of fruit flies with error pattern of deviation.

File Name: Supplementary Movie 2
Description: Tracking videos of fruit flies with error pattern of overlap.

File Name: Supplementary Movie 3
Description: Tracking videos of fruit flies with error pattern of mislocation.

File Name: Supplementary Movie 4
Description: Tracking videos of *Camponotus japonicus*.

File Name: Supplementary Movie 5
Description: Tracking videos of *Bombyx mori*.

File Name: Supplementary Movie 6
Description: Tracking videos of *Danio rerio*.
