# Supplementary material for: Large-language models facilitate discovery of the molecular signatures regulating sleep and activity
Source: Nat Commun. 2024 May 1;15:3685. doi: 10.1038/s41467-024-48005-w (PMC11063160; doi:10.1038/s41467-024-48005-w)
Supplement: Supplementary file 18 — Reporting Summary [file 41467_2024_48005_MOESM18_ESM.pdf]

nature portfolio

Corresponding author(s): Luoying Zhang, Yu Xue

Last updated by author(s): Mar 14, 2024

Reporting Summary

Nature Portfolio wishes to improve the reproducibility of the work that we publish. This form provides structure for consistency and transparency in reporting. For further information on Nature Portfolio policies, see our Editorial Policies and the Editorial Policy Checklist.

Statistics

For all statistical analyses, confirm that the following items are present in the figure legend, table legend, main text, or Methods section.

n/a	Confirmed	
☐	☒	The exact sample size (*n*) for each experimental group/condition, given as a discrete number and unit of measurement
☐	☒	A statement on whether measurements were taken from distinct samples or whether the same sample was measured repeatedly
☐	☒	The statistical test(s) used AND whether they are one- or two-sided<br>*Only common tests should be described solely by name; describe more complex techniques in the Methods section.*
☒	☐	A description of all covariates tested
☒	☐	A description of any assumptions or corrections, such as tests of normality and adjustment for multiple comparisons
☐	☒	A full description of the statistical parameters including central tendency (e.g. means) or other basic estimates (e.g. regression coefficient) AND variation (e.g. standard deviation) or associated estimates of uncertainty (e.g. confidence intervals)
☐	☒	For null hypothesis testing, the test statistic (e.g. *F*, *t*, *r*) with confidence intervals, effect sizes, degrees of freedom and *P* value noted<br>*Give P values as exact values whenever suitable.*
☒	☐	For Bayesian analysis, information on the choice of priors and Markov chain Monte Carlo settings
☒	☐	For hierarchical and complex designs, identification of the appropriate level for tests and full reporting of outcomes
☒	☐	Estimates of effect sizes (e.g. Cohen's *d*, Pearson's *r*), indicating how they were calculated

Our web collection on statistics for biologists contains articles on many of the points above.

Software and code

Policy information about availability of computer code

Data collection	Video capture and tracking analysis of fruit flies were performed as described. The commonly used biochemical incubators were purchased (Ningbo Yanghui Instrument Co., ltd.), and further adapted to accommodate video tracking instruments (Wuhan Yuda Instrument & Equipment Co., ltd.), which are infrared light camera with a wavelength of 850 nm. The video capture software package, AMcap (https://amcapdl.com/), was used for video tracking with a camera. Drosophila Activity Monitor system (Trikinetics) was used to analyze fly sleep by recording infrared beam breaks. StepOne Real-Time PCR Systems (Thermo Fisher Scientific) were used to conduct qRT-PCR experiment.
Data analysis	The video was converted into frame-by-frame image slices using a media transcoder OpenCV (https://opencv.org, Version 3.4.1) in Python (Version 3.5.6). The fly location in each image was analyzed with using Python (Version 3.8). The source codes of VTL and others used in this study have been deposited to GitHub (https://github.com/BioCUCKOO/VTL) with the DOI identifier (https://doi.org/10.5281/zenodo.10681859). SleepMat was used to analyze fly sleep in infrared-based acquisition systems. ImageJ was used to analyze distance among flies in social space assay. StepOne Software (v2.3, Applied Biosystems) was utilized to proceed and analyze qRT-PCR data.

For manuscripts utilizing custom algorithms or software that are central to the research but not yet described in published literature, software must be made available to editors and reviewers. We strongly encourage code deposition in a community repository (e.g. GitHub). See the Nature Portfolio guidelines for submitting code & software for further information.

Data

Policy information about availability of data

All manuscripts must include a data availability statement. This statement should provide the following information, where applicable:

- Accession codes, unique identifiers, or web links for publicly available datasets
- A description of any restrictions on data availability
- For clinical datasets or third party data, please ensure that the statement adheres to our policy

The processed video tracking data are available at https://vtl.biocuckoo.cn/download.html. The data of fly gene entries are downloaded from FlyBase (https://ftp.flybase.net/releases/FB2023_01/precomputed_files/genes/gene_map_table_fb_2023_01.tsv.gz), and the protein names are retrieved from UniProt (https://www.uniprot.org/uniprotkb?query=(taxonomy_id:7227). The data of GO annotation file (released on March 6, 2023) is obtained from the Gene Ontology Consortium (https://ftp.ebi.ac.uk/pub/databases/GO/goa/old/FLY/goa_fly.gaf.116.gz). The PPI data are retrieved from BioGRID (https://downloads.thebiogrid.org/File/BioGRID/Release-Archive/BIOGRID-4.4.202/BIOGRID-ALL-4.4.202.mitab.zip). The response data produced by GPT 3.5 and other remaining experimental data generated in this study are provided in the Supplementary Information/Source Data file.

Research involving human participants, their data, or biological material

Policy information about studies with human participants or human data. See also policy information about sex, gender (identity/presentation), and sexual orientation and race, ethnicity and racism.

Reporting on sex and gender	N/A
Reporting on race, ethnicity, or other socially relevant groupings	N/A
Population characteristics	N/A
Recruitment	N/A
Ethics oversight	N/A

Note that full information on the approval of the study protocol must also be provided in the manuscript.

Field-specific reporting

Please select the one below that is the best fit for your research. If you are not sure, read the appropriate sections before making your selection.

☒ Life sciences ☐ Behavioural & social sciences ☐ Ecological, evolutionary & environmental sciences

For a reference copy of the document with all sections, see nature.com/documents/nr-reporting-summary-flat.pdf

Life sciences study design

All studies must disclose on these points even when the disclosure is negative.

Sample size	Although there is no formal power calculation to define sample size, all cell and drosophila experiments were conducted independently with n =3+, and the sample size was chosen to be consistent with the previous literature by utilizing similar assays. Specific replicate numbers are indicated in the text.
Data exclusions	No data was excluded.
Replication	At least 3 times biological replicates were performed as described in the main text. In the analysis of "sleep latency", flies that didn't fall asleep overnight were not considered. Because "sleep latency" is defined that the time it takes a fly to fall asleep after light off during the night.
Randomization	Animals of the same genotype were sorted and collected into a common container, from which they were randomly selected for experiments.
Blinding	Investigators were not blinded to test groups during experiments because they are quantitative measurements that did not require subjective interpretation or judgement.

Reporting for specific materials, systems and methods

We require information from authors about some types of materials, experimental systems and methods used in many studies. Here, indicate whether each material, system or method listed is relevant to your study. If you are not sure if a list item applies to your research, read the appropriate section before selecting a response.

Materials & experimental systems

n/a	Involved in the study
☒	☐ Antibodies
☒	☐ Eukaryotic cell lines
☒	☐ Palaeontology and archaeology
☐	☒ Animals and other organisms
☒	☐ Clinical data
☒	☐ Dual use research of concern
☒	☐ Plants

Methods

n/a	Involved in the study
☒	☐ ChIP-seq
☒	☐ Flow cytometry
☒	☐ MRI-based neuroimaging

Animals and other research organisms

Policy information about studies involving animals; ARRIVE guidelines recommended for reporting animal research, and Sex and Gender in Research

Laboratory animals

All fly strains were obtained from Bloomington Drosophila Stock Center (BDSC), Vienna Drosophila Resource Center and TsingHua Fly Center or as gifts from colleagues. Fly strains used for genome-wide screen were listed in Supplementary Data 5. Most of the neurotransmitter related GAL4 lines were generated in Dr. Yi Rao's laboratory 47, including THGAL4, DATGAL4, Dop2RGAL4, Dop1R2GAL4, SerTGAL4, 5-HTR1BGAL4, TRHGAL4, AdoRGAL4, VGlutGAL4, GluRIAGAL4, ChATGAL4, Tdc2GAL4, Dop1R1GAL4, RdlGAL4, GABA-B-R1GAL4, GABA-B-R2GAL4 and GABA-B-R3GAL4. The Drosophila simulans w501 strain is a kind gift from Dr. Jian Lu at Peking University. The following fly lines were also used in this study: isogenic w1118 (BDSC:5905), DATfmn 25, Pdfrhan5304 (BDSC:33068), UAS-Cul3RNAi (11861R-2, Fly Stocks of National Institute of Genetics), UAS-CanA-14FRNAi (V30105), UAS-Fmr1 (BDSC:6928), UAS-NachBac (BDSC:9466), UAS-TrpA1 (BDSC:26263), UAS-mre11RNAi1 (THU5229), UAS-mre11RNAi2 (TH01614.N), UAS-NELF-BRNAi1 (THU0696), UAS-NELF-BRNAi2 (THU3523), UAS-NELF-BRNAi3 (THU4946), UAS-dcr2 (BDSC: 24650), elavGAL4 (BDSC:458), c305aGAL4 (BDSC:30829), c309GAL4 (BDSC:6906), 30YGAL4 (BDSC:30818), MB247GAL4 (BDSC:50742), c547GAL4 48, c819GAL4 (BDSC:30849), Dilp2GAL4 (BDSC:37516), Gad1GAL4 (BDSC:51630), VGATGAL4 (BDSC:58980), timGAL4 (BDSC:7126), cryGAL4-16 (BDSC:24514), 103YGAL4 (BDSC:30813), c42GAL4 (BDSC:30835), and Feb170GAL4 49. 3-5 day old male flies were used for all experiments except for those indicated otherwise. 6dpf zebrafish embryo of standard wild-type (AB) laboratory strain were used for behavior monitoring. Silk worms and ants were obtained from licensed private pet rearers with no additional information regarding strain and genetic background. The silk worms were fed fresh mulberry leaves and maintained under 12L12D at 25 °C with 70% relative humidity. The ants were kept in a dark plastic box in the incubator (25 °C and 60% relative humidity), and fed with honey and purified water with the weight ratio of 1:2. For behavior monitoring, first-instar silk worms and 4-month-old ants were used.

Wild animals

The study does not involve wild animals.

Reporting on sex

Both male and female flies were used when testing the video tracking system, and male flies were used for further studies because female flies may lay eggs which influence their activity. The other animals were only used for testing whether our video tracking system could be employed for monitoring activity in different species and thus effects of sex was not taken into consideration.

Field-collected samples

The study does not involve samples collected from the field.

Ethics oversight

All studies were approved by the Ethics Committee of Huazhong University of Science and Technology. All zebrafish maintenance procedures and experiments were in accordance with guidelines approved by the Ethics Committee of Huazhong University of Science and Technology (2019S907).

Note that full information on the approval of the study protocol must also be provided in the manuscript.
