# Supplementary figures and images for: Large-language models facilitate discovery of the molecular signatures regulating sleep and activity
Source: Nat Commun. 2024 May 1;15:3685. doi: 10.1038/s41467-024-48005-w (PMC11063160; doi:10.1038/s41467-024-48005-w)

## Slide 1
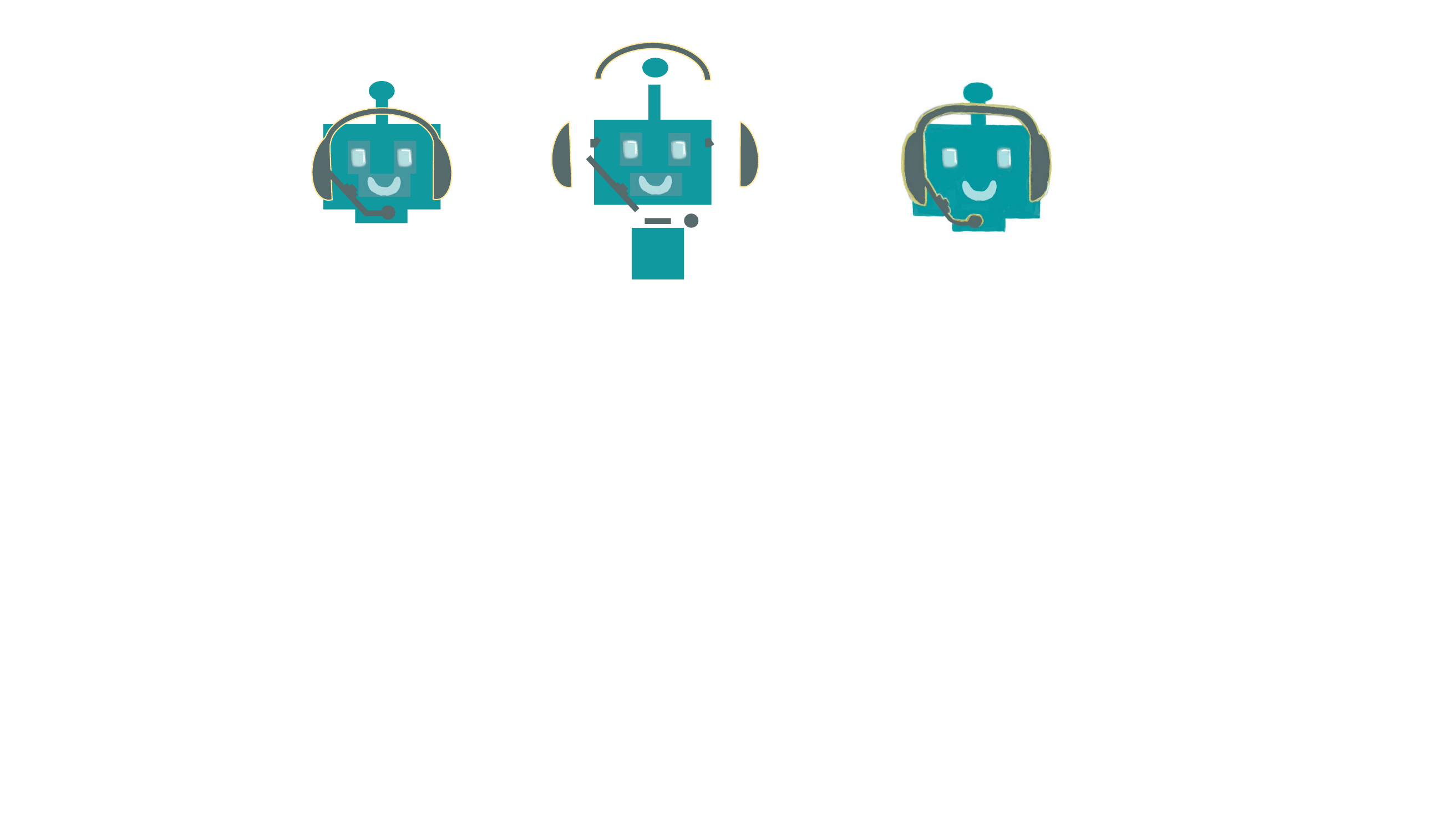

Supplement: Supplementary file 19 — Source Data [file 41467_2024_48005_MOESM19_ESM.zip › Source Data/Source files for icons/AI_icon.pptx]
